# Lola regulates *Drosophila *olfactory projection neuron identity and targeting specificity

**DOI:** 10.1186/1749-8104-2-14

**Published:** 2007-07-16

**Authors:** Maria Lynn Spletter, Jian Liu, Justin Liu, Helen Su, Edward Giniger, Takaki Komiyama, Stephen Quake, Liqun Luo

**Affiliations:** 1 Howard Hughes Medical Institute, Department of Biological Sciences, Stanford University, Stanford, California 94305, USA; 2 Howard Hughes Medical Institute, Department of Bioengineering, Stanford University, Stanford, California 94305, USA; 3 Department of Biomedical Engineering, Emory University, Atlanta, Georgia 30322, USA; 4 Janelia Farm Research Campus, Howard Hughes Medical Institute, Ashburn, VA 20147, USA; 5 National Institute of Neurological Disorders and Stroke, National Institutes of Health, Bethesda, MD 20892, USA

## Abstract

**Background:**

Precise connections of neural circuits can be specified by genetic programming. In the *Drosophila *olfactory system, projection neurons (PNs) send dendrites to single glomeruli in the antenna lobe (AL) based upon lineage and birth order and send axons with stereotyped terminations to higher olfactory centers. These decisions are likely specified by a PN-intrinsic transcriptional code that regulates the expression of cell-surface molecules to instruct wiring specificity.

**Results:**

We find that the loss of *longitudinals lacking *(*lola*), which encodes a BTB-Zn-finger transcription factor with 20 predicted splice isoforms, results in wiring defects in both axons and dendrites of all lineages of PNs. RNA *in situ *hybridization and quantitative RT-PCR suggest that most if not all *lola *isoforms are expressed in all PNs, but different isoforms are expressed at widely varying levels. Overexpression of individual *lola *isoforms fails to rescue the *lola *null phenotypes and causes additional phenotypes. Loss of *lola *also results in ectopic expression of Gal4 drivers in multiple cell types and in the loss of transcription factor gene *lim1 *expression in ventral PNs.

**Conclusion:**

Our results indicate that *lola *is required for wiring of axons and dendrites of most PN classes, and suggest a need for its molecular diversity. Expression pattern changes of Gal4 drivers in *lola*^-/- ^clones imply that *lola *normally represses the expression of these regulatory elements in a subset of the cells surrounding the AL. We propose that Lola functions as a general transcription factor that regulates the expression of multiple genes ultimately controlling PN identity and wiring specificity.

## Background

**Figure 1:**
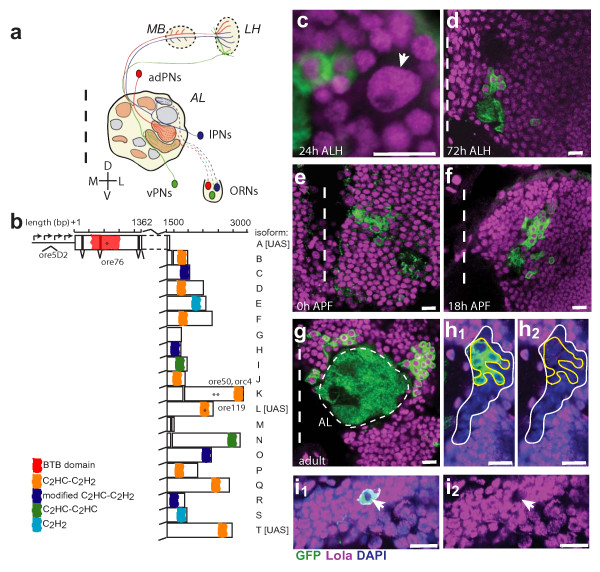
The *Drosophila *olfactory system, the *lola *gene, and Lola expression. **(a) **Schematic representation of the *Drosophila *antennal lobe. Olfactory receptor neuron (ORN) axons project to stereotyped glomeruli in the antennal lobe (AL). The anterodorsal (ad), lateral (l) and ventral (v) lineages of Gal4-GH146 positive PNs send dendrites to specific glomeruli in the AL and axons to specific regions in the mushroom body (MB) calyx and lateral horn (LH). **(b) **Schematic representation of the *lola *gene. *lola *is a complex genetic locus consisting of at least 20 isoforms generated by both *cis*- and *trans*-splicing of an amino-terminal BTB domain-containing common region to unique carboxy-terminal exons, most of which contain Zn fingers. Individual isoforms are labeled in accordance with previous nomenclature [16]. Mutations are marked by asterisks and isoforms with UAS transgenes are listed. **(c-g) **Time course of Lola expression using an antibody generated against the common region (amino acids 19–467). Lola is expressed in all neurons adjacent to the AL at all stages of development, shown at 24 h after larval hatching (ALH), 72 h ALH, 0 h after puparium formation (APF), 18 h APF and adult. Lola is also expressed in the neuroblast (arrow in c). In this and all subsequent figures, non-overlapping fluoresence channels are pseudocolored for ease of viewing: anti-CD8::GFP in green, anti-Lola in magenta. Dashed vertical line marks the midline. Antenna lobe (AL) outlined with thin dashed line in (g). **(h, i) **Specificity of anti-Lola antibody. Lateral *lola*^-/- ^(*ore76*) MARCM clone stained with anti-pan Lola. In (h), the white outline denotes entire MARCM clone determined by loss of Lola staining. The yellow outline denotes boundary of GH146 positive cells based on GFP expression. Loss of Lola staining in GH146 cells can be seen clearly in the magenta (Lola) only channel (h_2_). In (i), a DL1 single-cell *lola*^-/- ^MARCM clone shows a loss of Lola staining in the magenta channel (i_2_). The arrow marks the same cell in (i_1_) and (i_2_). Scale 10 μm. Green, anti-CD8::GFP; magenta, anti-Lola; blue, DAPI.

Nervous systems exhibit highly reproducible patterns of connectivity that are essential for their proper functions. Axon pathfinding in many systems is heavily dependent upon genetically programmed expression of guidance factors and their receptors [1]. Recent studies have indicated that dendrite target selection and aspects of synapse specificity can also be precisely genetically programmed in flies [2] and vertebrates [3]. For example, wiring specificity in the adult *Drosophila *olfactory system is achieved during pupal development before the onset of olfactory receptor expression [4]. Olfactory receptor neurons (ORNs) project their axons to glomeruli in the antennal lobe (AL), where they synapse with the dendrites of projection neurons (PNs) (Figure 1a; reviewed in [5,6]). PNs target their dendrites to single glomeruli and send their axons to stereotypic locations in the mushroom body (MB) and lateral horn (LH) according to their glomerular class [7-9]. Most PNs are derived from three neuroblast lineages, anterodorsal (adPNs), lateral (lPNs) and ventral (vPNs). adPNs and lPNs innervate intercalating but non-overlapping sets of glomeruli, suggesting lineage-specific control of targeting. Additionally, adPNs are specified by birth order, suggesting that instructive information within a lineage determines wiring patterns [2]. Indeed, PN dendritic patterning precedes ORN axon patterning: by the time pioneering ORN axons arrive at the developing AL, PN dendrites have already formed a coarse map by virtue of their specific dendritic targeting [4].

Several cell-surface proteins, including Sema-1a, Dscam and N-Cadherin, have been shown to play different roles in PN dendritic development. These studies suggest a model in which PN dendrites first target to a rough region of the AL based on molecular gradients and then are further refined by dendro-dendritic and dendro-axonal interactions [10-12]. The expression of these and additional cell-surface proteins are likely controlled by a transcriptional code that acts to uniquely specify the wiring aptitude of individual PNs[13,14]. Studies of several transcription factors (TFs) support the existence of a transcriptional hierarchy in PNs. Some factors, such as the LIM cofactor Chip, appear to affect wiring in all PN classes [14]. Other TFs show lineage specific restriction in expression and regulatory effects. For example, the LIM-homeodomain TF Islet is required for proper targeting of a subset of adPNs and lPNs, while the homeodomain TF Cut is required in only a subset of lPNs and all vPNs [14]. As another example, POU-domain TFs Acj6 and Drifter have restricted expression patterns in adPN and lPN lineages, respectively, and control wiring specificity in their respective lineages [13]. A recently identified BTB-Zn-finger TF, Chinmo, regulates birth order-dependent wiring of adPNs. Loss of *chinmo *results in adPNs born in early larval life acquiring the targeting specificity of late-born PNs within the same lineage [15]. There are also TFs that appear to affect targeting of a single PN type. For example, the LIM-homeodomain TF Lim1 is necessary for proper targeting of a single vPN to the DA1 glomerulus, and is regulated by Cut. The Zn-finger TF Squeeze appears to be necessary for the innervation of a single lPN glomerulus, DM5 [14]. All these studies support a model where the targeting specificity of a particular PN is regulated by a unique complement of TFs, and additional members of the TF code remain to be identified.

The gene *longitudinals lacking *(*lola*) encodes a molecularly diverse BTB-Zn-finger TF with at least 20 unique protein isoforms (Figure 1b). Each isoform is formed by combining a set of common BTB-containing amino-terminal exons to unique Zn-finger-containing carboxy-terminal exons via *trans*- and/or *cis*-pre-mRNA splicing [16-18]. The BTB (Broad complex, Tramtrack, Bric à brac) domain, also referred to as the POZ (poxvirus and Zn-finger) domain, is a common domain likely involved in protein-protein interactions [19]. Most *lola *isoforms have one or more unique Zn-fingers of either the C_2_H_2 _type that binds DNA, or the unusual C_2_HC class that binds nucleosomes, non-histone proteins, RNA and DNA [20,21]. At least some Lola isoforms bind DNA directly [22]. Interestingly, three Lola isoforms lack Zn-fingers and theoretically could be involved in heteromeric regulatory interactions with other Lola isoforms, as Lola was found to bind itself in yeast-two-hybrid interactions and co-immunoprecipitation experiments [21,23]. Lola also likely interacts with other proteins such as chromosomal kinase JIL-1 [21].

Lola was initially identified as a factor that regulates axon guidance in the embryonic central nervous system longitudinal tracks, and is reported to function in a wide array of other cellular processes. *lola *mutants exhibit defects in the extension of embryonic longitudinal axons and midline crossing, orientation of lateral chordotonal neurons, and ISN_b _axon growth and elaboration [24,25]. Mutation of Lola isoforms K or L is sufficient to inactivate a specific subset of *lola *functions in ISN_b _neurons, suggesting that different Lola isoforms may have unique functions [16]. *lola *was recently identified to disrupt ORN axonal innervation of the AL in an overexpression screen [26]. *lola *may exert its effect through transcriptional regulation of cell-surface molecules, and has been reported to genetically interact with *Notch*, *slit*, and *robo *[27,28]. *lola *may have a more general regulatory role as a polycomb group (PcG) factor affecting cell proliferation via the Notch pathway and regulating wing development through a genetic interaction with *cut *[29].

In this study, we show that *lola *plays an essential role in PN identity and wiring specificity. *lola *appears to be a general factor that affects wiring of both axons and dendrites in all three lineages of PNs. Overexpression of *UAS-lola T *and *UAS-lola L*, but not *UAS-lola A*, results in wiring defects. Additionally, expression of single *lola *isoforms is insufficient to rescue the *lola *null phenotype and often causes additional defects specific to the isoforms expressed, suggesting the importance of Lola molecular diversity in regulating PN wiring specificity. Indeed most *lola *isoforms are expressed in PNs but at different levels. Finally, consistent with previous findings of transcriptional regulation and potential PcG function, we find that *lola *likely regulates *lim1*and several Gal4 enhancer trap lines in PNs. We suggest that *lola *regulates PN wiring through transcriptional regulation of downstream targets involved in defining neuronal identity and targeting specificity.

## Results and discussion

We investigated the function of *lola *in wiring specificity of olfactory PNs by using the MARCM (mosaic analysis with a repressible cell marker) system [30]. PNs send dendrites to specific glomeruli in the AL [2] and axons to the MB and LH in highly stereotyped patterns [7-9] (Figure 1a), allowing us to examine *lola *function in both dendrites and axons.

### Lola is expressed in projection neurons and neuroblasts

Using a specific antibody raised against the domain common to all Lola isoforms (Figure 1b), we found that Lola is expressed in all cells in the AL region at all stages of development from larva to adult (Figure 1c–g). These include expression in post-mitotic cells such as PNs, but also in neuroblasts, which we can identify by their size during larval development (Figure 1c). Antibody specificity was confirmed by loss of Lola staining in neuroblast and single-cell MARCM clones homozygous for a *lola *null allele (*ore76*, which introduces an early stop codon in the BTB domain of all Lola isoforms (Figure 1b), hereafter referred to as *lola*^-/-^) in adult (Figure 1h,i) and in early pupa (data not shown). Note that Lola staining can be used to identify all mutant cells in a MARCM clone that are not necessarily labeled by *UAS-mCD8::GFP *because Lola is expressed ubiquitously throughout the brain, while Gal4 enhancer traps often have a more limited domain of expression (Figure 1h).

### *lola *mutant projection neurons have dendrite wiring defects

**Figure 2:**
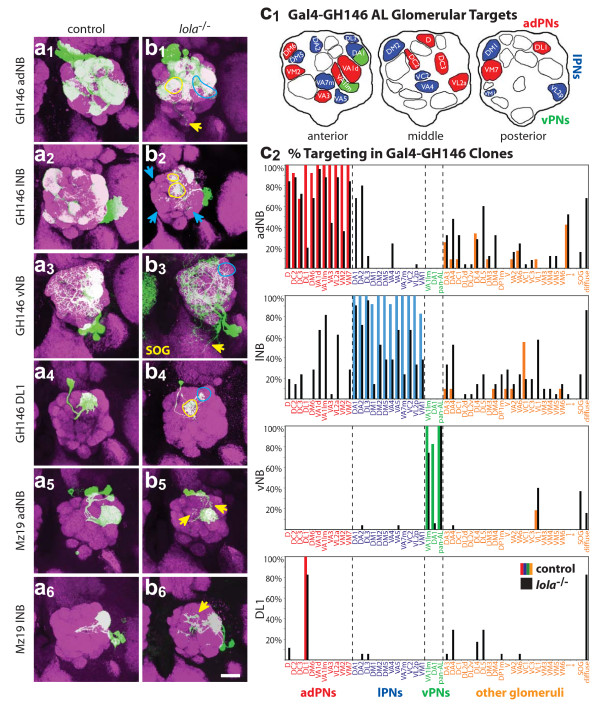
Dendritic targeting defects in *lola*^-/- ^MARCM clones. Representative confocal images of **(a) **wild-type control and **(b) ***lola*^-/- ^anterodorsal (1), lateral (2), or ventral (3) MARCM neuroblast (NB) clones or DL1 single cell (SC) clones (4) labeled with Gal4-GH146, and anterodorsal (5) and lateral (6) neuroblast clones labeled with Gal4-Mz19. Blue arrows and circles demark a loss of correct targeting, while yellow arrows and circles demark off-target innervation. Both anterodorsal and ventral *lola*^-/-^clones have ectopic dendritic extensions to the suboesophageal ganglion (SOG). anti-CD8::GFP in green, anti-nc82 neuropil in magenta. Scale bar, 20 μm. **(c_1_) **Normal targets of Gal4-GH146 in anterior, middle and posterior sections of the AL. Red denotes dorsal target, blue denotes lateral target, and green denotes ventral target. **(c_2_) **Quantification of adNB, lNB, vNB and DL1 clone phenotypes. The percentage of clones targeting a particular glomerulus is denoted on the Y-axis, while individual glomeruli are listed on the X-axis and grouped according to lineage by color as in (c_1_). Glomeruli in orange are not normally targeted by Gal4-GH146 positive PNs by heatshocking at 24 h ALH. Control clone innervation is denoted by colored bars corresponding to lineage, and *lola*^-/- ^clone innervation is denoted by black bars. Note the decrease of correct targeting and increase of off-target innervation in *lola*^-/- ^brains for all clone types. Additionally, the final 'diffuse' bar quantifies the number of brains that exhibit diffuse and wandering dendrites that fail to specifically innervate glomeruli.

Since Lola is expressed in PNs during AL development, and mutant clones effectively eliminate Lola protein during the period of dendritic targeting, we proceeded to analyze the phenotype of *lola*^-/- ^PN MARCM clones. Using the Gal4-GH146 enhancer-trap line to label PNs, we observed wiring defects in all lineages of PN MARCM clones as well as anterodorsal single cell clones that normally target the DL1 glomerulus (Figure 2).

In wild type, Gal4-GH146 labels PNs of three neuroblast lineages. PNs from adPN and lPN lineages target their dendrites to stereotypical, intercalating but non-overlapping sets of glomeruli [2] (Figure 2a1,a2,c1). In general, phenotypes of *lola*^-/- ^adPNs and lPNs can be classified into two main categories: a loss of targeting to normal target glomeruli and a gain of targeting to off-target (ectopically targeted) glomeruli (Figure 2b1,b2). However, the phenotypes are highly variable between brains and there are few consistent 'shifts' where one off-target glomerulus is innervated in place of a normal target. The phenotypes also have variable penetrance, with innervation of both normal and ectopic targets ranging from partial to full innervation from brain to brain. Specifically, *lola*^-/- ^adPNs often invade DA1, DL3 and VA4, which are normal lPN targets. Likewise, mutant lPNs often innervate VA1d, VA1lm, DM6, D, DC2 and VM7, which are normal adPN targets (Figure 2c2). *lola*^-/- ^dendrites also appear more diffuse in general, and often 'wander' through the lobe, wrapping around, but not actually innervating, glomeruli. Additionally, *lola*^-/- ^dendrites are frequently not restricted to the AL and innervate regions of the suboesophageal ganglion (SOG; Figure 2b1,b3,c2). Wild-type vPNs strongly innervate DA1 and VA1lm, and one vPN (pan-AL PN) innervates the entire AL (Figure 2a3). *lola*^-/- ^vPNs show a strong loss of AL restriction and nearly 37% show ectopic dendritic extensions to the SOG (Figure 2b3,c2). Additionally, DA1 targeting in ventral clones is almost completely lost (6% versus 82% in wild type) and VA1lm targeting is reduced nearly 30% (Figure 2c2). We also analyzed an independent *lola*^*ore*5*D*2 ^allele of *lola*, which is predicted to be strongly hypomorphic due to a P-element insertion in the *lola *promoter (Figure 1b). *lola*^*ore*5*D*2 ^clones display many of the phenotypes observed in the *ore76 *null allele (see additional file 1), indicating that the phenotypes we observed are caused by loss of *lola*.

Given that Lola is ubiquitously expressed, dendritic defects in neuroblast clones can be attributed to its requirement in the neuroblast or post-mitotic cells or both; furthermore, one cannot determine cell autonomy with certainty. We therefore examined single cell clones that target to the DL1 glomerulus to test whether *lola *has a cell-autonomous and post-mitotic function. We found that nearly 80% of *lola*^-/- ^DL1 single cell clones show targeting defects (Figure 2b4,c2) and 23% completely fail to innervate DL1. Another 54% innervate DL1 (though often weakly or partially) and have additional extensions either wandering between glomeruli or, more frequently, innervating glomeruli anterior to DL1 in the AL (see Table S1 in additional file 2). Using the pan-Lola antibody, we can verify there are no unlabeled clones in the vicinity of the AL and be certain we are looking at cell-autonomous effects. At least in the DL1 PN, therefore, *lola *appears to have post-mitotic, cell intrinsic effects on dendritic targeting.

To extend higher resolution phenotypic analysis to other PN classes, we examined adPN and lPN neuroblast clones using Gal4-Mz19, which labels a small subset of GH146-positive neurons that project to DA1, VA1d and DC3 [4]. Interestingly, dendritic targeting of *lola*^-/- ^DA1 and VA1d PNs exhibit phenotypes with much less severity and penetrance than other *lola*^-/- ^PNs. Normal target glomeruli were almost always strongly targeted, and defects mainly consisted of additional dendritic extensions into the AL (Figure 2b5,6). Consistent with this, we find that DA1 (innervated by lPNs) and VA1d (innervated by adPNs) are two of the most 'stable' glomeruli and show little phenotype in GH146 mutant neuroblast clones (Figure 2c2). These two glomeruli are also often ectopically innervated by *lola*^-/- ^PNs from the opposite lineage (that is, DA1 is ectopically targeted by *lola*^-/- ^adPNs while VA1d is ectopically targeted by *lola*^-/- ^lPNs). This experiment suggests that distinct PN classes differentially require *lola*.

We also tested available mutations that affect individual *lola *isoforms. The *orc4 *(affecting isoform K; Figure 1b) allele did not show obvious defects (see additional files 1, 4 and 8). The *ore119 *allele (affecting isoform L; Figure 1b) shows a small subset of the phenotypes observed in *lola*^-/- ^(see additional file 1). *lola L*^-/- ^adPN targeting appears normal, but lPNs show a loss of targeting to VM1 and DA2, while ventral clones show a loss of DA1 targeting. However, we cannot rule out the possibility that an additional mutation on the *lola L *mutant chromosome contributes to the phenotype, as our attempts to generate a second allele specific for *lola L *have not been successful and the *ore119 *phenotype could not be rescued by a *UAS-lola L *transgene (data not shown). In fact, MARCM expression of *lola L *in PNs results in severe phenotypes by itself (see below).

### *lola *mutant projection neurons have axon wiring defects

**Figure 3:**
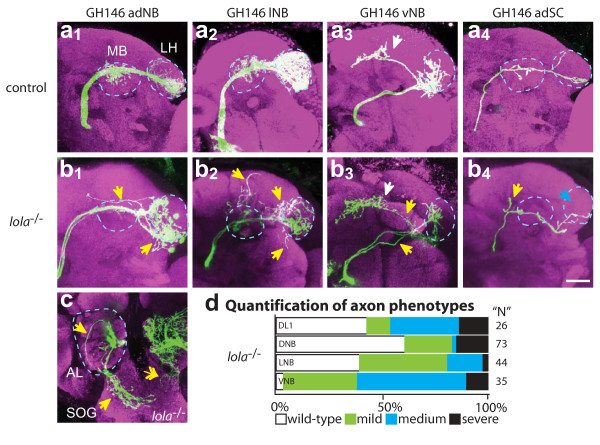
Axon wiring defects in *lola*^-/- ^MARCM clones. Representative confocal images of **(a) **wild-type control and **(b) ***lola*^-/- ^adNB (1), lNB (2) vNB (3) and DL1 SC (4) clones showing PN axons near their normal target: the mushroom body (MB) and lateral horn (LH), both marked by blue dotted circles. **(c) **Example of an axon mistargeting to SOG after exiting the posterior AL (blue dotted circle). Double-headed white arrow demarks dendritic mistargeting to SOG. Blue arrows denote a lack of correct targeting while yellow arrows demark ectopic branching. anti-CD8::GFP in green, anti-nc82 neuropil in magenta. Scale bar, 20 μm. **(d) **Quantification of severity of axon targeting defects. Mild: axons target correctly but have ectopic branches (b_1_, b_2_); medium: axons target to the correct region but follow an incorrect trajectory or bifurcate (b_3_, b_4_); severe: axons target to the SOG or abnormal brain regions (c).

In addition to dendritic phenotypes, we observed defects in PN axonal wiring in *lola*^-/- ^MARCM clones. As with dendrites, defects were found in adPNs, lPNs and vPNs as well as DL1 single-cell clones (Figure 3). Phenotypes were highly variable both in penetrance and severity in neuroblast as well as single cell clones. In a semi-quantitative approach, we scored phenotypes as mild, medium and severe (see Figure 3 legend). A separate quantification of the targeting defects limited by specific phenotypes (ectopic branching, misrouting of axons, SOG targeting and a lack of MB/LH innervation) is presented in Table S2 (see additional file 2).

In *lola*^-/- ^adPNs and lPNs, the most common defect was ectopic branching outside of the region of the MB or the LH (28% and 63%, respectively; Figure 3b1,2; see Table S2 in additional file 2). Some clones showed a lack of innervation, where innervation of the MB failed to occur or branch extension in the LH was severely limited. A smaller percentage of *lola*^-/- ^clones (15% adPNs and 7% PNs) showed medium or severe axon defects (Figure 3d). Hypomorphic clones (*ore5D2*) show similar, but mostly mild, targeting defects (see Table S2 in additional file 2).

Axons of vPNs are the most severely affected: nearly 100% of clones exhibit defects in both *lola*^-/- ^and hypomorphic (*ore5D2*) alleles (Figure 3b3,d; see Table S2 and S4 in additional file 2). Often ventral axons show several defects in the same brain. Seventy-nine percent of *lola*^-/- ^clones show ectopic branching defects (often extensions from the LH towards the SOG or an increase in branches along the medial extension). Thirty-four percent of clones show misrouting defects; most often a single axon defasciculates from the bundle and projects directly to the medial-central region of the LH instead of the ventral region (compare Figure 3a3 to 3b3). Nine percent of null clones and 47% of hypomorph clones show clear axonal targeting to the SOG (this may be higher in the null but it can be difficult to distinguish between axon and dendrite mistargeting unless clear defasciculation from the main axon bundle is visible). We observe that all PN axons exit the AL in a single bundle, but then some axons defasciculate and project ventrally to the SOG (Figure 3c). It is interesting to note that axons misguided to the SOG typically project to similar locations in the SOG, suggesting they may be responding to some cues in this region.

More than 50% of DL1 single cell clones show axon defects (Figure 3b4,d, see Table S3 in additional file 2), suggesting that Lola acts cell-autonomously in axon targeting. Twenty-six percent of DL1 *lola*^-/- ^axons have ectopic branches, often with additional branches in the LH region (see Table S2 in additional file 2). Fifteen percent of clones fail to extend branches into the MB (see Table S2 in additional file 2). Some DL1 axons clearly bifurcate just prior to entering the MB. It is interesting to note that many DL1 clones that fail to target the DL1 glomerulus have relatively normal axonal projections. A severe dendrite phenotype does not predict the most severe axon phenotypes (or vice versa), suggesting that these two processes are separable, in agreement with recent observations of independence between axon and dendrite targeting [14]. These results suggest that *lola *has post-mitotic, cell-autonomous functions in PN axon targeting.

### Analysis of *lola *isoform expression by *in situ *hybridization and RT-PCR

Our MARCM analysis suggests that *lola *has an important function for PN dendritic and axonal targeting. Given that *lola *encodes 20 alternatively spliced isoforms, several scenarios may account for *lola *function (see additional file 3). In scenario I, different isoforms may be expressed in different neuroblast lineages and thereby specify their lineage-specific wiring analogous to POU transcription factors [13]. In scenario II, individual PNs may express a unique isoform or combination of isoforms independent of lineage to specify PN identity. In scenario III, all cells may express most or all *lola *isoforms.

**Figure 4:**
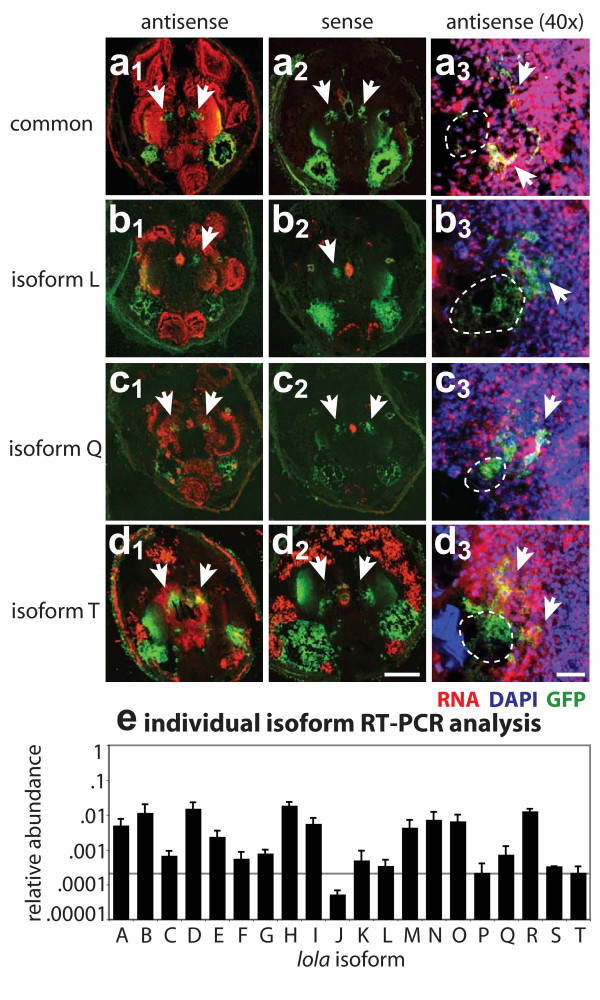
Expression of *lola *isoforms in the *Drosophila *brain. **(a-d) **RNA *in situ *analysis of *lola *isoforms. (a_1_) An antisense probe generated against the common region of *lola *labels uniformly throughout the brain at 0 h APF. (a_2_) A sense control to the same probe shows little specific staining. (a_3_) A magnified view of the AL reveals *lola *expression in all PN cell bodies. PN cell bodies are marked by white arrows, while dotted while lines demark the rough area of the AL neuropil in each section that is not stained by DAPI. Midline to the left, lateral to the right. Isoform specific probes to isoform L (b), isoform Q (c) and isoform T (d) show different patterns of expression throughout the brain at 0 h APF, while sense control probes (b_2_-d_2_) show little specific labeling. Closer inspection of AL regions at a higher magnification (b_3_-d_3_) reveals that most isoforms appear to be expressed in PNs. Scale bars: 20 μm (a_3_-d_3_); 200 μm (a_1_-d_2_). DIG-labeled RNA probe in red, DAPI in blue, GFP in green. See Additional file 2 for *in situ *analysis of additional *lola *isoforms and additional labeling of section morphology. **(e) **Quantitative RT-PCR of laser-captured PN enriched-samples verifies *in situ *results that most isoforms are expressed in PNs. Additionally, different *lola *isoforms are expressed at different levels at 0 h APF, with about 100-fold difference between highest and lowest expression levels. Data are displayed by *lola *isoform on the X-axis and by relative abundance on a log scale on the Y-axis, where relative abundance has been determined against the level of *actin42 *expression. Error bars represent the standard deviation from four independent samples, and each sample was tested independently five times per device. Horizontal solid line represents a confidence limit of the average relative expression of samples near the detection limit based on CT values and reproducibility.

To distinguish between these possibilities, we performed RNA *in situ *hybridization with probes against 16 of the 20 *lola *isoforms at 0 h after puparium formation (APF), a developmental time when PNs start to elaborate dendrites in the AL [4]. We first verified our *in situ *protocol by using probes against *drifter *(*dfr*), a TF that has previously been shown to be expressed in GH146 labeled lPNs but not adPNs [13]. As expected, we found that signal from anti-sense probe against *dfr *mRNA coincides with green fluorescent protein (GFP)-labeled lPNs but not adPNs (see additional file 3). A sense control probe shows little signal coinciding with the GFP-positive cell bodies (see additional file 3). We then performed *in situ *analysis with *lola *probes. A probe to the *lola *common region shows expression throughout the brain (Figure 4a1,a3) compared with sense control (Figure 4a2), consistent with our Lola antibody staining results. For individual isoform mRNAs, we detect signal in the region of the AL for all *lola *isoforms tested, as well as other brain regions. Some isoforms have narrower domains of expression, such as isoforms N (see additional file 3) and T (Figure 4d1,d3), which have much stronger signal in the central brain and weaker staining in the optic lobe. Other isoforms appear to be expressed more uniformly throughout the region of the AL (Figure 4; see additional file 3). It should be noted that several isoforms have more punctate signal that co-localizes with the nucleus (see additional file 3f, k, r), perhaps indicative of pre-mRNA. Another caveat is that mRNA production may not translate to protein expression, as recently shown by the wide mRNA but narrow protein expression domains of *nerfin *[31]. We did not find PN lineage-specific expression for any of the *lola *isoforms, nor is there evidence that any isoforms are expressed in a subset of PNs within a lineage. Rather, most *lola *isoform mRNAs appear to be expressed in all PNs, consistent with scenario III (see additional file 3).

As an independent and more quantitative method of assessing expression of different isoforms in PNs, we performed laser-dissection microscopy (LCM) to capture GH146-positive PNs from frozen sections of 0 h APF tissue, followed by quantitative RT-PCR to detect the expression level of individual isoforms. Although we do not have single-cell resolution, laser-dissection allows us to assay mRNA expression in a small subset of neurons. Consistent with the *in situ *hybridization data, at 0 h APF we detected most *lola *isoforms by RT-PCR, although several isoforms are near the RT-PCR detection limit (Figure 4e). We find marked differences (up to 100-fold) in the level of RNA expression of individual isoforms in PNs at 0 h APF (Figure 4e). We additionally analyzed AL samples from different developmental timepoints (third instar larvae, 0 h APF, 25 h APF and adult) and find that expression levels of several *lola *isoforms, for example isoform E, H, I, L, M and Q, appear to be developmentally regulated (see additional file 4). Our *in situ *results also suggested that *lola *isoforms are expressed at different levels in the optic lobe. Indeed, RT-PCR on LCM captured optic lobe at 0 h APF confirmed that mRNAs of different *lola *isoforms are expressed at different levels and revealed differences in the pattern of isoform expression between AL and optic lobe samples (see additional file 4). Together, these data suggest that the level of individual *lola *isoform expression is dynamically regulated and that individual isoforms may function at different stages in development.

### *UAS-lola *transgene overexpression phenotypes

**Figure 5:**
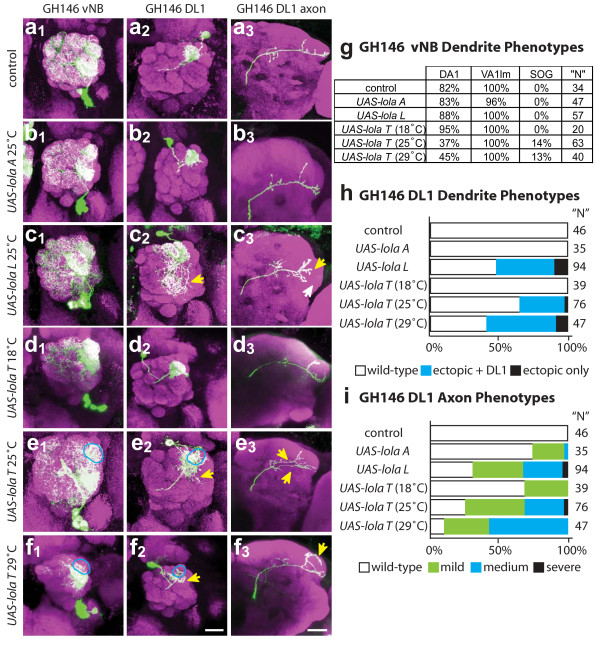
Effects of overexpressing different *lola *isoforms on dendritic and axonal phenotypes. Representative confocal images of **(a) **control and **(b-f) **Gal4-GH146 MARCM overexpression phenotypes in vNB (1), DL1 (2) and DL1 axon (3). *UAS-lola A *(b) overexpression shows little defects, whereas *UAS-lola L *(c) and UAS-*lola T *(d-f) show defects in most classes of PNs. The effect of UAS-*lola T *(d-f) expression increases with increasing temperature. anti-CD8::GFP in green, anti-nc82 neuropil in magenta. Scale bar, 20 μm. Blue circles demark a loss of correct targeting, while yellow arrows demark off-target innervation or branching. **(g) **Quantification of vNB targeting phenotypes. Data are presented as percentage of observed clones that innervate a given target. Overexpression of *UAS-lola T *at 25°C or 29°C results in a decrease in DA1 targeting and an increase in ectopic dendritic extensions to the SOG. **(h, i) **Quantification of DL1 phenotypes. Dendrite defects are quantified (h) by percent of clones that fail to target DL1 and those that target DL1 but have additional dendrite extensions and ectopic innervations in the AL. Axon defects (i) are quantified as in Figure 3.

Genetic analysis using available isoform-specific alleles (see additional file 1) indicates that at least two isoforms (K and L) are not required for dendritic targeting of the majority of PN classes. To test further potential functions of different isoforms, we examined the overexpression phenotypes of three *lola *isoforms. Previous reports suggested differences between the overexpression phenotypes of *lola A *and *lola T *in motor neurons [25]. We have generated a third transgene, *UAS-lola L*. We found that Gal4-GH146 expression of any of these three UAS transgenes results in lethality, but we bypassed this lethality by MARCM mediated transgene expression in specific neuroblast lineages or single cells (Figure 5; see additional file 5). We tested one, three and two insertions for *UAS-lola A*, *UAS-lola L *and *UAS-lola T*, respectively (see Table S1 in additional file 2). Levels of transgene expression were verified in our attempted rescue experiments (see below). Because Gal4-GH146 drives transgene expression in post-mitotic PNs (J Liu, MS, LL, unpublished observation), the overexpression phenotypes we describe below are caused by post-mitotic expression of *lola *transgenes.

Neuroblast or single cell clones overexpressing *UAS-lola A *(which lacks a Zn-finger) do not exhibit detectable defects (Figure 5b; see additional file 5). However, *UAS-lola L *and *UAS-lola T *(which both have C_2_HC-C_2_H_2_-type Zn-fingers) show strong targeting defects in adPN and lPN neuroblast clones (see additional file 5). Phenotypes often include a large increase in wandering dendrites in the AL and some degree of loss of targeting and ectopic targeting. Often dendrites appear to be largely restricted to one region of the AL such as dorsal or lateral, and have wandering dendrites through this region in many glomeruli, but fail to innervate normal targets or extend any dendrites into other regions of the AL (see Additional file 5c, e, f). *UAS-lola T *expression also results in a decrease in the number of labeled PNs, in part likely due to cytotoxicity based on the observation of clones with very punctate GFP staining patterns indicative of cell and process degeneration, although we were unable to reliably stain brain tissues for activated caspase-3 (V Trunova and E Giniger, unpublished data). Due to the difficulty in quantifying phenotypes in adPN and lPN neuroblast clones because of the magnitude and variability, we focused our analysis on vPN neuroblast clones and single-cell DL1 clones.

vPN neuroblast clones in animals raised at 25°C show no defects when overexpressing *lola A *(Figure 5b1) and weak phenotypes when overexpressing *lola L *(Figure 5c1), but vPNs are strongly affected by *lola T *overexpression (Figure 5e1,f1). Targeting to DA1 is often lost in vPN clones overexpressing *lola T *(Figure 5e1,f1,g). vPN dendrites frequently extend outside of the AL boundary, most often in the ventral region projecting towards the SOG. Processes of the pan-AL vPN are not elaborated properly and only partially innervate the AL.

DL1 single-cell clones were used to examine cell-autonomous overexpression effects on both dendritic and axonal targeting. *lola A *overexpression did not affect targeting of either DL1 axons or dendrites (Figure 5b2,b3). The DL1 dendrites targeted correctly with *lola L *overexpression, but frequently showed a large increase in dendritic mass outside of DL1, wandering throughout the AL (Figure 5c2,h). In axons, overexpression of *lola L *often resulted in an increase in branching in MB and LH regions (Figure 5c3,i). In the LH, these additional branches resulted in a loss of the stereotypic 'L' projection and a failure of branches to fully extend into normal target areas. *lola T *overexpression resulted in variable DL1 phenotypes, ranging from a complete loss of DL1 innervation and targeting to other regions of the AL to DL1 targeting with dendritic extensions outside of the DL1 glomerular region (Figure 5e2,f2,h). *lola T *overexpression also affected axon targeting, most often resulting in a high degree of ectopic branching, axon bifurcation and incorrect LH innervation patterns (Figure 5e3,f3,i). It is interesting to note that isoforms L and T are normally expressed at low levels based on RT-PCR results, while isoform A is expressed roughly 10-fold higher (Figure 4e). Differences in overexpression phenotype may be related to the presence or absence of a Zn-finger domain, and/or to the expression level relative to the endogenous level of expression.

### *UAS-lola T *dosage sensitivity

In the course of our overexpression study, we tested two different insertions of *UAS-lola T*. We noticed that the insertion on the third chromosome gives stronger and more penetrant phenotypes than the insertion on the X chromosome, and hypothesized that this was due to differences in transgene expression levels. To test this hypothesis, we altered the levels of *UAS-lola T *(X insertion) transgene expression by raising animals at 18°C, 25°C or 29°C, as levels of Gal4-induced transgene expression increase with increasing temperatures. In neuroblast clones we observed a qualitative increase of phenotypic severity in both dendritic and axonal targeting with increasing temperature (Figure 5d,e,f). This dosage sensitivity can be quantified with the DL1 dendritic phenotypes (Figure 5h). Flies raised at 18°C have few dendritic phenotypes, while at 25°C and 29°C dendrites mistarget and have ectopic innervations and axons have additional branches and bifurcations (Figure 5h,i; see Table S1 in additional file 2). Additionally, dosage sensitivity is evident in vNB DA1 and SOG extension phenotypes (Figure 5g). This experiment indicates that PN dendritic targeting is sensitive to the expression level of a specific *lola *isoform.

### Individual *lola *isoform expression in a *lola *null background

**Figure 6:**
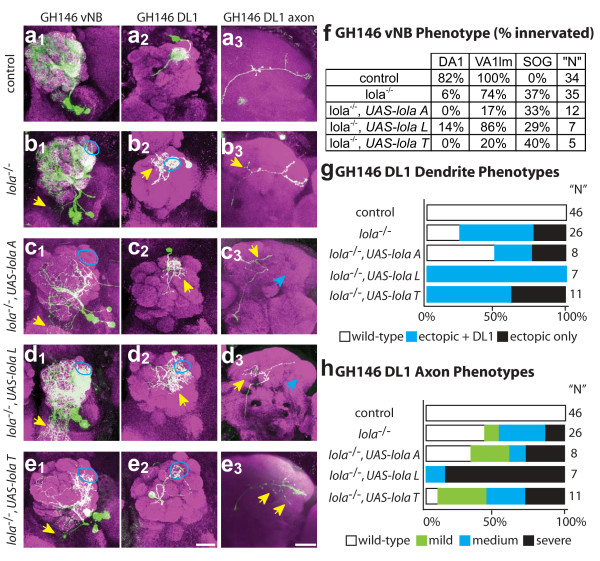
UAS-transgene overexpression in *lola*^-/- ^MARCM clones results in additive dendritic and axonal targeting defects. Representative images of **(a) **control and **(b-e) **experimental conditions as indicated for the vNB (1), DL1 single-cell dendrite (2) and axon (3) phenotypes of *UAS-lola A *(c), *UAS-lola L *(d) and *UAS-lola T *(e) expression in *lola*^-/- ^MARCM clones. None of the transgenes are able to rescue the *lola*^-/- ^phenotype. Expression of either *UAS-lola A *and *UAS-lola T *both result in a decrease in dendrite elaboration. **(f) **Quantification of vNB phenotypes. Data are presented as percentage of observed clones that innervate a particular target. *UAS-lola *transgenes fail to rescue the *lola*^-/- ^phenotype. Often transgene expression has an additive effect and further disrupts normal targeting, for example, there are dramatic losses of VA1lm targeting with *UAS-lola A *and *UAS-lola T*. 'N' for rescue experiments are low due to extreme difficulty in generating MARCM UAS-expression clones in a *lola*^-/- ^background. **(g) **Quantification of DL1 dendrite phenotypes as in Figure 5. *UAS*-*lola L *and *UAS-lola T *clones lack normal dendritic targeting. **(h) **Quantification of DL1 axonal phenotypes as in Figure 2. *UAS-lola L *and *UAS-lola T *show increases in axonal defects compared with *lola*^-/- ^alone. anti-CD8::GFP in green, anti-nc82 neuropil in magenta. Scale bar, 20 μm.

To test if individual *lola *isoforms can rescue any of the *lola*^-/- ^phenotype, we performed MARCM analysis on clones that are simultaneously *lola*^-/- ^and express either *UAS-lola A*, *UAS-lola L *or *UAS-lola T *transgenes. We verified all transgenes are expressed based on the presence of Lola antibody staining in these otherwise *lola*^-/- ^clones. Expression of all transgenes was qualitatively similar at a level roughly half as intense as neighboring non-mutant cells expressing endogenous levels of Lola (data not shown). Instead of rescuing the *lola*^-/- ^phenotype, overexpression of transgenes in mutant PNs resulted in additive defects (Figure 6; see additional file 6). In adPN and lPN neuroblast clones, simultaneous loss of endogenous *lola *and expression of any of the single isoforms caused striking additive effects in cell loss and lack of dendritic extension, which are described in detail in additional file 6. Below we focus on vPN neuroblast clones and DL1 single cell clones.

Additive effects of transgene expression were most striking in vPNs. *lola*^-/- ^vPNs expressing *UAS-lola A*, *UAS-lola L *or *UAS-lola T *resulted in strong reduction of VA1lm and DA1 targeting (Figure 6f). Additionally, the pan-AL neuron often showed little dendritic process elaboration and rarely innervated much of the AL. Dendrites and axons of ventral cells often looked punctate, indicative of degeneration. These data indicate that expression of UAS transgenes in *lola*^-/- ^clones fails to rescue *lola *phenotypes and further disrupts dendritic wiring. Additionally, expression of certain *lola *isoforms alone may be toxic, perhaps due to dominant-negative interactions with other proteins or *lola *dimerization.

DL1 clones were difficult to obtain for all *UAS*-*lola *transgenes expressed in *lola*^-/- ^clones, possibly due to toxicity that we observe in neuroblast clones. Phenotypes did not always appear additive for DL1 clones, perhaps due to low levels of MARCM-based expression in single cell clones during the critical time of dendritic and axonal targeting. The strongest dendritic phenotypes were observed in *lola*^-/- ^cells expressing *UAS-lola T*, where every clone either failed to innervate DL1 or had extensions into other regions of the AL (Figure 6e,g). *lola*^-/- ^cells expressing *UAS-lola L *innervated DL1 in addition to having extensive dendritic innervation in the rest of the AL, while *lola*^-/- ^cells expressing *UAS-lola A *were variable, with half appearing normal (Figure 6c,d,g). All *lola*^-/- ^cells expressing a *UAS-lola *transgene showed axonal defects, ranging from a failure to extend or elaborate branches in the LH to an increase in ectopic branching (Figure 6h). These phenotypes suggest that individual *lola *isoforms can function post-mitotically to disrupt dendrite and axon targeting in DL1, and suggest that Lola interacts either as homomers or with other BTB domain proteins. Additionally, expression of single *lola *isoforms, especially *lola T*, appears to be toxic to many cells, so it is unclear if phenotypes are directly related to targeting or perhaps secondary effects due to disruption of cell viability.

Taken together, these results suggest that isoform diversity of the *lola *locus is important. Post-mitotic expression of a single isoform is not sufficient to rescue the *lola *phenotype, and indeed is more disruptive to a cell than simple loss of *lola*. This does not, however, rule out an additional function of Lola in the neuroblast. Lastly, some aspects of Lola function are likely mediated through the BTB domain alone, as additive phenotypes can be caused by expressing isoforms with or without Zn-fingers.

### Evidence that *lola* regulates cell identity and transcription

The dendritic and axonal phenotypes in *lola*^-/-^clones we described so far could be caused by the action of Lola on PN targeting directly (for instance, by regulating the expression of cell surface receptors), or Lola could regulate PN identity with axonal and dendritic mistargeting as a secondary consequence. Although these models are not mutually exclusive, we provide some evidence in *lola*^-/-^MARCM clones using several Gal4 lines, including Mz19, GH146 and NP3529, to support the second hypothesis.

**Figure 7:**
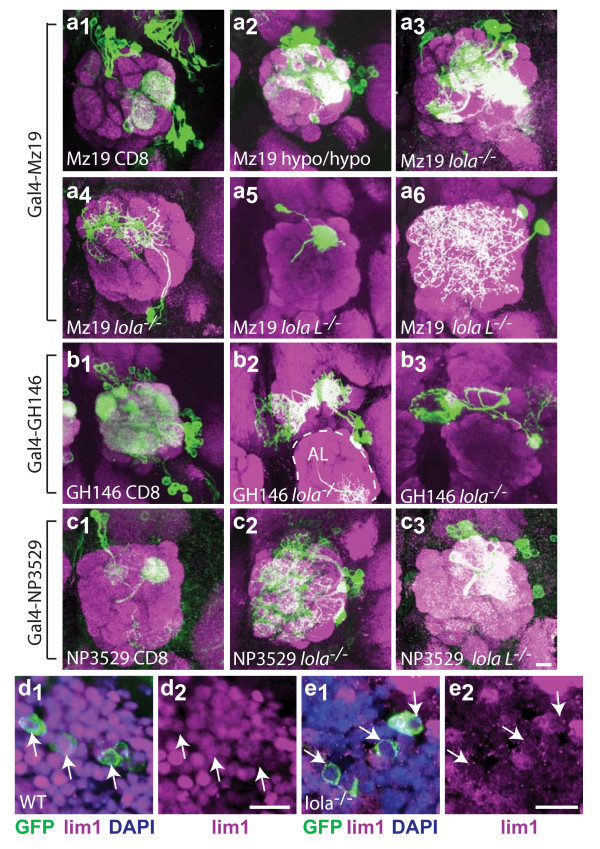
Evidence that *lola *regulates expression of Gal4 drivers and Lim1. **(a) **Gal4-Mz19 mislabeling analysis. Whole-animal Gal4-Mz19, *UAS-GFP::CD8 *expression labels a small subset of PNs (a_1_). See Figure 2 for Mz19 and GH146 MARCM controls. Mutations in *lola *result in the labeling of more cell bodies than Mz19 normally labels (hypo: ore5D2) (a_2_, a_3_), as well as the labeling of ectopic cell types such as local interneurons (LNs) (a_4_, a_6_) and DL1 that is normally labeled by Gal4-GH146 but not Gal4-Mz19 (a_5_). **(b) ***lola*^-/- ^Gal4-GH146 clones frequently mislabel a set of neurons in the region of the AL that project to the medial lobe of the MB and to the central body complex. **(c) **NP3529 mislabeling analysis. NP3529 labels two dorsal PNs that project to DL1 (c_1_). *lola*^-/- ^clones dramatically increase the number of cells labeled near the AL, including cells in the lateral lineage and LNs (c_2_). *lolaL*^-/- ^clones show similar mislabeling (c_3_). anti-CD8::GFP in green, anti-nc82 neuropil in magenta. **(d, e) ***lola *regulates Lim1 expression in vPNs. Wild-type cells in the region of the antennal lobe, including most (at least four) vPNs express Lim1 (d). In *lola*^-/- ^cells, Lim1 expression is lost in vPNs (e). Note that the *lola*^-/-^clone includes neighboring cells in addition to those labeled by Gal4-GH146, and many cells have lost Lim1 staining. White arrows mark the cell bodies of GFP positive cells. anti-CD8::GFP in green, anti-Lim1 in magenta, DAPI in blue. Scale bar, 10 μm.

MARCM with several mutant alleles suggests that *lola *regulates the expression of Gal4-Mz19. Ten percent of *lola*^*ore*5*D*2 ^(*lola *hypomorph) and *lola*^-/- ^clones show a 30% to 50% increase in the number of labeled PNs and targeted glomeruli (Figure 7a2,a3; MARCM control Figure 2a5,a6). Strikingly, we observed (in 64% of all adPN clones) that *lola L*^-/-^(*lola*^*ore*119^) clones frequently target the DL1 glomerulus (Figure 7a5), which is normally targeted by GH146 positive, but never Mz19 positive, PNs. As our heat-shock timing in these experiments should not result in any Mz19 single cell clones (they are born later) but coincides exclusively with the birth of the DL1 PN [2], the simplest interpretation is that Mz19 is now misexpressed in DL1 PNs. This would suggest that *lola *normally represses Mz19 expression in DL1 PNs. As additional evidence that *lola *regulates gene expression, 25% of clones of both *lola*^-/- ^and *lola L*^-/-^visualized by Gal4-Mz19 appear to label local interneurons (LNs), an additional cell-type that innervates a large number of glomeruli in the AL and lacks axonal projections out of the AL region (Figure 7a4,a6). The similarity of isoform L specific phenotypes to phenotypes in other *lola *alleles suggests that *lola*^ore119 ^phenotypes in PNs might be caused by the disruption in isoform L instead of a second site mutation on the same chromosomal arm. Thus, it appears that in the absence of Lola (particularly the Lola L isoform), Gal4-Mz19 is ectopically expressed in neuronal types that normally do not express this Gal4 enhancer trap line.

Gal4-GH146, used extensively in this study, labels a large subset of PNs in the AL, roughly 50 adPNs, 35 lPNs and 5 or 6 vPNs (Figure 7b1; MARCM controls Figure 2a1–3). However, in *lola*^-/- ^neuroblast clones we observe labeling of a new cell type that innervates a small region of the AL and sends projections to the medial axonal lobe of the MB as well as the central body complex (Figure 7b2–3). These cells are never labeled in control experiments, but make up 22% of all observed clones in *lola*^-/- ^experiments (see Table S5 in additional file 2). Interestingly, we observe similar projection patterns in wild-type neurons labeled with other Gal4 drivers, suggesting that central body complex targeted *lola*^-/- ^clones are a different cell type where Gal4-GH146 expression is normally repressed by *lola*.

Gal4-NP3529 labels two dorsal PNs that project to DL1 (Figure 7c1). Clones generated with both *lola*^-/- ^and *lola L*^-/- ^label much larger subsets of cells. These clones often contain PNs that innervate typical GH146 target glomeruli, as well as LNs and other PN classes that do not express GH146. *lola*^-/- ^clones label many additional cells in the adPN lineage as well as lPNs and LNs (Figure 7c2). *lola L*^-/- ^clones show similar types of mislabeling (Figure 7c3). This strongly suggests that *lola *function is normally necessary to repress the expression of NP3529 in multiple cell types.

Lola also appears to positively regulate gene expression. Lim1 is expressed in a subset of PNs, including most (at least four) vPNs labeled by GH146 (Figure 7d1,d2) [14]. In *lola*^-/- ^mutant cells, Lim1 expression is lost (Figure 7e1,e2). Lim1 expression cannot be rescued by expression of either *UAS-lola A *or *UAS-lola T *in *lola*^-/- ^clones (n = 2 each for *UAS-lola A *and *UAS-lola T*; data not shown). Lim1 expression is also lost in vPNs mutant for the TF *cut *[14]. However, Cut expression is not altered in *lola*^-/-^clones, and Lola expression is not altered in *cut*^-/-^clones (data not shown), suggesting these TFs coordinately regulate Lim1 expression through parallel pathways. Intriguingly, PNs mutant for *lola*, *lim1 *or *cut *all fail to target DA1, suggesting that *cut *and *lola *may be necessary for regulation of other TFs as well as wiring. Antibody staining patterns of other TFs previously identified to be important for PN targeting, including *acj6*, *drifter*, *cut*, *chip *and *islet*, are not disrupted in *lola*^-/- ^clones (data not shown). Taken together, these data suggest that *lola *can positively and negatively regulate gene expression of other genes known to label subsets of PNs or to play a role in PN wiring specificity.

## Conclusion

Wiring specificity in *Drosophila *olfactory projection neurons is regulated by intrinsic TFs [13-15]. Here we have characterized an additional TF, Lola, as necessary for proper AL wiring specificity of both PN dendrites and axons. *lola*^-/- ^mutant clones show a wide variety of wiring phenotypes, including a loss of targeting to correct glomeruli, innervation of ectopic targets, a loss of lineage restriction in dendritic projections, a loss of AL boundary restriction, and an increase in wandering projections and ectopic branch formation. Additionally, *lola *appears normally to repress the expression of multiple Gal4 drivers in certain cell-types and positively regulate *lim1 *in vPNs. These data suggest that *lola *regulates multiple developmental processes, including identity as well as wiring specificity of PNs, consistent with its expression in neuroblasts and post-mitotic neurons. In fact, the wide variety of phenotypes observed in *lola *mutants may be a general characteristic of genes involved in multiple developmental processes, as similar phenotypes are observed in mutants of general TF complex cofactors, such as *chip *[14], or are known chromatin regulatory genes (D Berdnik and LL, unpublished data). This is the first report linking roles for *lola *in both fate and wiring specificity in the same cell-type, suggesting that PNs have a tightly linked relationship between targeting specificity and cell identity.

*lola *is a highly complex genetic locus and encodes at least 20 different splice isoforms [16]. Although Lola is expressed throughout development, quantitative RT-PCR reveals that individual isoforms have different levels of expression. The diversity of *lola *isoform expression in PNs also appears important, as expression of a single *UAS*-*lola *transgene fails to rescue null mutant phenotypes and often results in even more severe phenotypes that are specific to the expressed *lola *isoform. We tested available isoform-specific *lola *alleles, and found that an isoform L (but not K) mutant has mild targeting defects. The difficulties of generating other isoform-specific alleles in this locus, coupled with the strong gain-of-function effect of expressing transgenes of a single isoform, made it difficult for us to investigate further isoform-specific functions of *lola *in PN development. However, taken together with previous reports [16,25,16,25], our results support the notion that different isoforms may have unique functions in PNs in addition to embryonic motor neurons.

Several lines of evidence suggest that Lola can regulate chromatin structure. Loss of function (LOF) mutations in *lola *enhance the Pc/+ phenotype and result in a significant increase in the number of sex-combs on the second leg, as do other known PcG factors [28]. This reported PcG interaction, in addition to direct binding of the chromosomal kinase JIL-1 and the presence of the nucleosome binding C_2_HC class Zn-finger [20,21], suggests that at least some Lola isoforms may be part of chromatin regulatory complexes. Additionally, at least one Lola isoform has been shown to bind directly to DNA and regulate expression of the *copia *retrotransposon [22]. Many chromatin regulators are integral components in pathways of cell fate specification. Neuronal cell fate is closely linked with dendritic and axonal targeting, particularly in PNs where cell identities based on birth order are manifested as wiring choices and target specification [2]. This link between targeting and cell identity has been suggested in the MB, where mutation of the chromatin modifier *polyhomeotic *causes MB neurons to randomly express certain *Hox *genes that are normally suppressed and to display a wide variety of axon targeting defects that are neither a complete targeting fate switch nor consistent between individuals [32]. This is reminiscent of the *lola *phenotype in PNs, suggesting that *lola *might function in a similar manner.

*lola *may additionally play a more direct role in targeting. *lola *mutants affect some glomeruli more severely than others and have post-mitotic functions in DL1 dendritic targeting, suggesting that there is a differential requirement for, or sensitivity to, the loss of *lola *between individual PNs. *lola *also has specific targeting phenotypes in the embryonic central and peripheral nervous systems, as mutations in *lola *seem to disrupt axon guidance and extension of the Ap^+^, VUM, and ISN_b _neurons without affecting the numbers or differentiation of these neuronal subtypes [16,24,25,29]. Taken together, this evidence suggests that *lola *may participate both in fate determination and, more directly, in wiring specificity. If the processes of fate determination and wiring specificity are separable, one might expect a factor that controls wiring to directly regulate the expression of genes that function in targeting, such as cell surface receptors, while a factor that controls cell fate should affect the expression of 'fate' markers that subsequently disrupt downstream expression of the components of the wiring machinery in an indirect fashion. Future identification of direct targets of *lola *regulation will be informative and necessary to understand the exact mechanism of *lola *function in PNs.

The idea that TFs can differentially control fate specification and wiring specificity is consistent with the model of a hierarchical TF code that determines wiring specificity. It is interesting to consider the possibility that some TFs may be used at multiple points during identity and wiring specification. In neuron sub-type specification in the ventral nerve cord, the TF *collier *specifies the precursors of the Ap neuron subset, but must be turned-off in three of the four Ap cells as subsequent participation of *collier *in a multi-protein complex later in development defines a unique sub-type of Ap neurons [33]. During development of the peripheral nervous system, *cut *specifies sensory neuron identity and is required again later in development at different levels in subpopulations of dendritic arborization neurons to specify subclass arborization [34]. Similarly, in vPN development, the TF Cut is necessary in the neuroblast for generation of the correct number of vPNs and postmitotically for specific targeting of the VA1lm glomerulus [14]. Temporal regulation and multi-functionality of TFs are possible mechanisms to limit the required number of genes in a TF code while still uniquely specifying cell identity. Lola is a potential candidate for a TF that may function in this way, both specifying cell identity and playing more specific roles later in development in regulating wiring specificity.

## Methods

### Fly stocks and reagents

MARCM stocks used were as previously published [35]. Gal4-GH146 and Gal4-Mz19 are available from the Bloomington Stock Center, and Gal4-NP3529 is from the Kyoto Stock Center. lola^ore76 ^(early stop codon in BTB domain predicted to be protein null for all Lola isoforms), lola^ore119 ^(Pro712 to Leu mutation in the Zn-finger linker region of isoform L predicted to reduce DNA binding by 95%), lola^orc4 ^(C to T transition introduces an early stop in amino acid 771 of isoform K), lola^5*D*2 ^(P-element insertion into the *lola *promoter predicted to be strongly hypomorphic allele and eliminate most *lola *transcription), *UAS-lola A *(X), *UAS-lola T *(X) and *UAS-lola T *(III) have been described previously [16,24,25]. We generated three new *UAS-lola L *lines with insertions on chromosome III. The *lola L *coding region was cloned into p [UAST] [36] and injected into embryos. Single G_0 _males were crossed to yw; Pin/CyO virgins. Single F_1 _males with red eyes were crossed to yw; Pin/CyO virgins to establish stocks and identify the insertion chromosome.

Genotypes of flies generated in experiments are as follows: (Lola protein expression time course and Lim1 WT staining) yw; Gal4-GH146, *UAS-mCD8a::GFP *(GH146 staining and LOF analyses) hsFlpCD8; FRT42D, Gal80/FRT42D, lola^x^, GH146, *UAS-mCD8a::GFP *[where x denotes any *lola *allele] (control GH146 MARCM) hsFlpCD8; FRT42D, Gal80/FRT42D, GH146, *UAS-mCD8a::GFP *(lola^ORC4 ^LOF analysis) hsFlpCD8; FRT42D, Gal80/FRT42D, lola^orc4^, GH146, *UAS-mCD8a::GFP *(control Mz19 MARCM) hsFlpCD8; Gal4-Mz19, *UAS-mCD8a::GFP*, FRT42D, Gal80/FRT42D (Mz19 LOF analysis) hsFlpCD8; Gal4-Mz19, *UAS-mCD8a::GFP*, FRT42D, Gal80/FRT42D, lola^x ^(*UAS-lola A *misexpression) *UAS-lola A*/hsFlp^122^, *UAS-mCD8a::GFP*; FRT42D, Gal80/FRT42D, Gal4-GH146, *UAS-mCD8a::GFP *(*UAS-lola L *(X) misexpression) *UAS-lola T*/hsFlp^122^, *UAS-mCD8a::GFP*; FRT42D, Gal80/FRT42D, Gal4-GH146, *UAS-mCD8a::GFP *(*UAS-lola T *(III) misexpression) hsFlp^122^, *UAS-mCD8a::GFP*; FRT42D, Gal80/FRT42D, Gal4-GH146, *UAS-mCD8a::GFP*; *UAS-lola T *(*UAS-lola L *misexpression) hsFlp^122^, *UAS-mCD8a::GFP*; FRT42D, Gal80/FRT42D, Gal4-GH146, *UAS-mCD8a::GFP*; *UAS-lola L *(*UAS-lola A *rescue) *UAS-lola A*/hsFlp^122^, *UAS-mCD8a::GFP*; FRT42D, Gal80/FRT42D, lola^ORE76^, Gal4-GH146, *UAS-mCD8a::GFP *(*UAS-lola T *rescue) *UAS-lola T*/hsFlp^122^, *UAS-mCD8a::GFP*; FRT42D, Gal80/FRT42D, lola^ORE76^, Gal4-GH146, *UAS-mCD8a::GFP *(*UAS-lola L *rescue) hsFlp^122^, *UAS-mCD8a::GFP*; FRT42D, Gal80/FRT42D, lola^ORE76^, Gal4-GH146, *UAS-mCD8a::GFP*; *UAS-lola L *(NP3529 LOF analysis) hsFlp^122^, *UAS-mCD8a::GFP*;FRT42D, Gal80/FRT42D, lola^x^;Gal4-NP3529.

### Clonal and phenotypic analysis

MARCM was performed as previously described [30,35]. Clones were induced at 24 h APF for all Gal4 driver lines. Brains were dissected, fixed in 4% paraformaldehyde for 20 minutes at room temperature and stained. Brains were mounted in Slow-Fade Gold or BioMedia Mounting Medium and imaged on a Zeiss LSM Meta 510 system. Z-series were captured in 1 μm sections. Fluorophores were imaged using band-pass filters to remove cross-detection between channels and pseudocolored for ease of viewing. Images were processed and scored using Image J [37] or Zeiss software. Data were analyzed using FileMaker Pro and Microsoft Excel. In all, we scored 48 distinct glomeruli in the AL, based on published models [38]. Glomeruli were considered innervated when dendrites entered the nc82 stained synaptic dense region and elaborated extensions within the glomerulus (that is, a single extension passing through a glomerulus was not enough to warrant scoring as positively innervated).

### Antibodies and staining

Antibody staining was performed as previously described [14,39]. The following primary antibodies were used; rat anti-mCD8a (Caltag [Burlingame, California, USA]), 1:100; monoclonal antibody (mAb) nc82 (developed by E Buchner and obtained from the Developmental Studies Hybridoma Bank (DSHB) [Iowa City, Iowa, USA]), 1:30; rabbit anti-Lola common region [24], 1:100; mAb anti-Cut (DSHB), 1:20; guinea pig anti-Lim1 (a gift from J Botas), 1:500; rat anti-Islet (a gift from J Skeath), 1:1,000; rabbit anti-Chip (a gift from D Dorsett), 1:500; mouse monoclonal anti-Acj6 [40], 1:5; rat anti-Drifter [41], 1:3,000; mouse monoclonal 7F1 anti-lola zf5 [21]. Nuclei were counterstained with DAPI (5 mg/ml stock 1:1,000).

### Laser-dissection microscopy and RT-PCR

White y, w; Gal4-GH146, UAS-CD8::GFP pre-pupa (considered 0 h APF) were collected, covered with optimal cutting temperature compound (Tissue Tek O.C.T. #4583 [Sakura Finetek, Torrance, California, USA]), frozen on dry ice and stored at -80°C not longer than 1 month. Blocks were sectioned on a cryostat and sections containing ALs were mounted on PEN membrane slides (Leica #11505158) and stored on dry ice. Slides were put through an EtOH dehydration series (75%, 95%, 100% EtOH 5 minutes each), treated for 2 minutes with xylenes, dried and mounted for LCM capture. PN enriched samples as identified by GFP fluorescence were immediately captured into PCR tube caps on a Leica LCM microscope (model ASLMD) using Laser Microdissection software version 4.4. Tubes were frozen on dry ice and stored at -80°C up to one day. RNA was extracted using the Arcturus PicoPure™ RNA Isolation kit (#KIT0204, Mountain View, California, USA) as per the manufacturer's instructions.

The cRNA samples were used as the template for another round of reverse transcription (#18080-051, Invitrogen [Carlsbad, California, USA]), producing a high yield of cDNA. The cDNA samples were aliquoted and stored at -20°C before use. Unique primer pairs and Taqman probes were designed for individual *lola *isoforms. The Taqman probes were synthesized by Integrated DNA Technologies, Inc. (Coralville, Iowa, USA). Microfluidic matrix chips (48 × 48 arrays, Fluidigm (South San Francisco, California, USA) were used to perform real time PCR assays in a high throughput fashion. Each chip allows complete combinatorial tests (48 × 48 = 2,304, N = 48) between N independent cDNA samples and N distinct primer pairs [42]. A separate paper is to be published with more technical details.

### *In situ *hybridization

Probes for *lola *were designed to the common region and isoform specific exons (for primers see Table S6 in additional file 2). Isoforms are referred to based on the previously described naming scheme [16]. Probes were amplified from adult genomic preps or from embryo RNA via RT-PCR, topo-cloned and sequenced to verify identity. DIG-labeled RNA probes were generated from plasmid DNA using the Roche DIG-RNA Labeling Kit (#11175025910, Indianapolis, Indiana, USA) following the manufacturer's protocol. Sense and antisense probes were generated from the same plasmid, taking advantage of the SP6 and T7 dual promoter flanked PCR2.1-Topo (# K460001, Invitrogen) cloning site. Probes were diluted to 10 ng/μl and stored at -80°C for up to 6 months. We collected 0 h APF pre-pupa, which were frozen in OCT on dry ice, stored at -80°C not more than 1 month and sectioned on a cryostat. An in-depth protocol for *in situ *hybridization is available in additional file 7. Briefly, slides were fixed in 4% PFA for 15 minutes, washed, acetylated, washed, and pre-hybridized at room temperature. Probes were added at concentrations ranging from 5 ng to 100 ng and slides were incubated 18 h overnight at 55°C. Slides were washed, native biotin blocked and blocked in normal goat serum. Sheep anti-DIG or rabbit anti-DIG and chicken anti-GFP primaries were added and incubated overnight at 4°C. Slides were washed, treated with a tyramide signal amplification kit for DIG signal detection (Dako #K0620 [Carpinteria, California, USA]), then incubated with secondary anti-chicken antibody. Slides were washed, mounted and imaged.

## Competing interests

S Quake is a co-founder, consultant, and equity holder of Fluidigm Corporation. All other authors declare that they have no competing interests.

## Authors' contributions

Maria Spletter designed the study, wrote the manuscript, and performed or participated in all experiments. Justin Liu participated in Lola antibody staining experiments, Gal4-GH146 driver characterization at larval time-points, and MARCM experiments. Helen Su helped develop the *in situ *protocol and participated in *in situ *hybridization experiments. Jian Liu helped conceive of and design and participated in LCM RT-PCR experiments. Stephen Quake helped conceive of and participated in the design and analysis of LCM RT-PCR experiments. Edward Giniger helped revise the manuscript and provided initial reagents and intellectual input for the study. Takaki Komiyama originally conceived of the study, performed initial PN phenotype characterization of the *lola*^*ORE*5*D*2 ^allele and helped revise the manuscript. Liqun Luo participated in the overall design and coordination of the study and helped write the manuscript. All authors have read and approved the final manuscript.

## Supplementary Material

Additional file 1 Additional *lola *mutant allele MARCM analysis demonstrating dendritic targeting defects. Supplemental Figure S1 showing targeting defects in *lola*^*ore*5*D*2^, *lola*^*orc*4^, and *lola*^*ore*119 ^mutant PNs. Click here for file

Additional file 2 Supplemental Tables S1-S6. Supplemental tables and legends referenced in the main text. This includes a semi-quantitative analysis of axon targeting defects in *lola*^-/- ^and *lola*^*ORE*5*D*20-/-^, a complete summary of DL1 dendrite and axon phenotypes by allele as percentage affected, a complete summary of vPN dendritic phenotypes by allele as percentage targeted correctly, clonal frequencies for MARCM experiments and primer sequences for *in situ *hybridization probes. Click here for file

Additional file 3 Additional *lola *isoform *in situ *in the *Drosophila *brain. Supplemental Figure S2 showing controls and verification for our *in situ *technique and additional *lola *isoform hybridization results. Click here for file

Additional file 4 Additional *lola *isoform RT-PCR analysis in the *Drosophila *brain. Supplemental Figure S3 showing addition LCM RT-PCR experiments comparing *lola *isoform expression at different developmental time points and in different brain tissues. Click here for file

Additional file 5 Effects of *UAS*-*lola *overexpression on adPN and lPN dendrites and axons. Supplemental Figure S4 showing adPN and lPN phenotypes in MARCM clones in a wild-type background expressing *UAS-lola A*, *UAS-lola L *and *UAS-lola T*. Click here for file

Additional file 6 adNB and lNB phenotypes in *lola*^-/-^, *UAS*-*lola *MARCM clones. Supplemental Figure S5 showing adPN and lPN phenotypes in *lola*^-/- ^clones expressing *UAS-lola A*, *UAS-lola L *and *UAS-lola T*. Click here for file

Additional file 7 Protocol for RNA *in situ *hybridization in fly larval/pupal/adult tissues. Our detailed protocol for performing RNA *in situ *hybridization on *Drosophila *at later developmental time points. Click here for file

Additional file 8 Legends for supplemental Figures S1-S5. All supplemental figure titles and legends. Click here for file
