# Supplementary figures and images for: Lola regulates Drosophila olfactory projection neuron identity and targeting specificity
Source: Neural Dev. 2007 Jul 16;2:14. doi: 10.1186/1749-8104-2-14 (PMC1947980; doi:10.1186/1749-8104-2-14)

# Figure S1

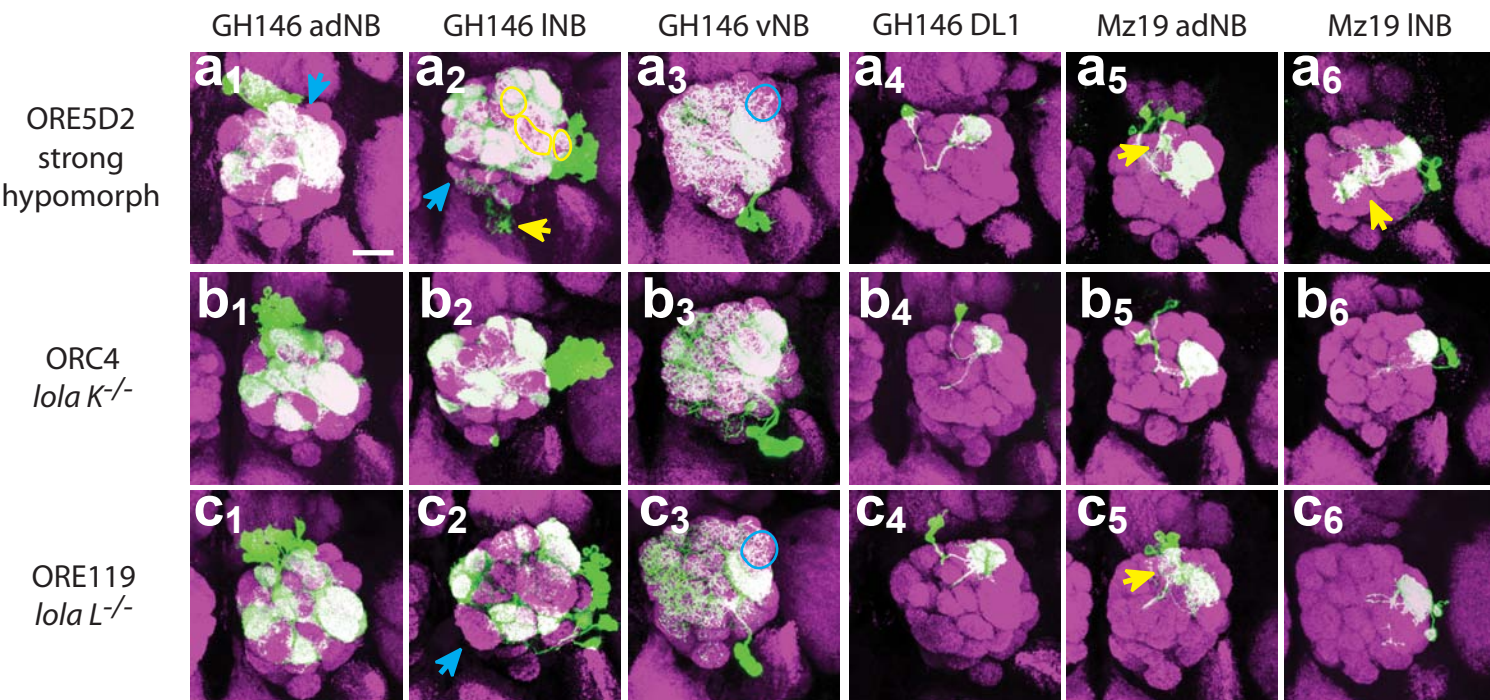

Supplement: Additional file 1 — Additional lola mutant allele MARCM analysis demonstrating dendritic targeting defects. Supplemental Figure S1 showing targeting defects in lolaore5D2, lolaorc4, and lolaore119 mutant PNs. [file 1749-8104-2-14-S1.pdf]

# Figure S2

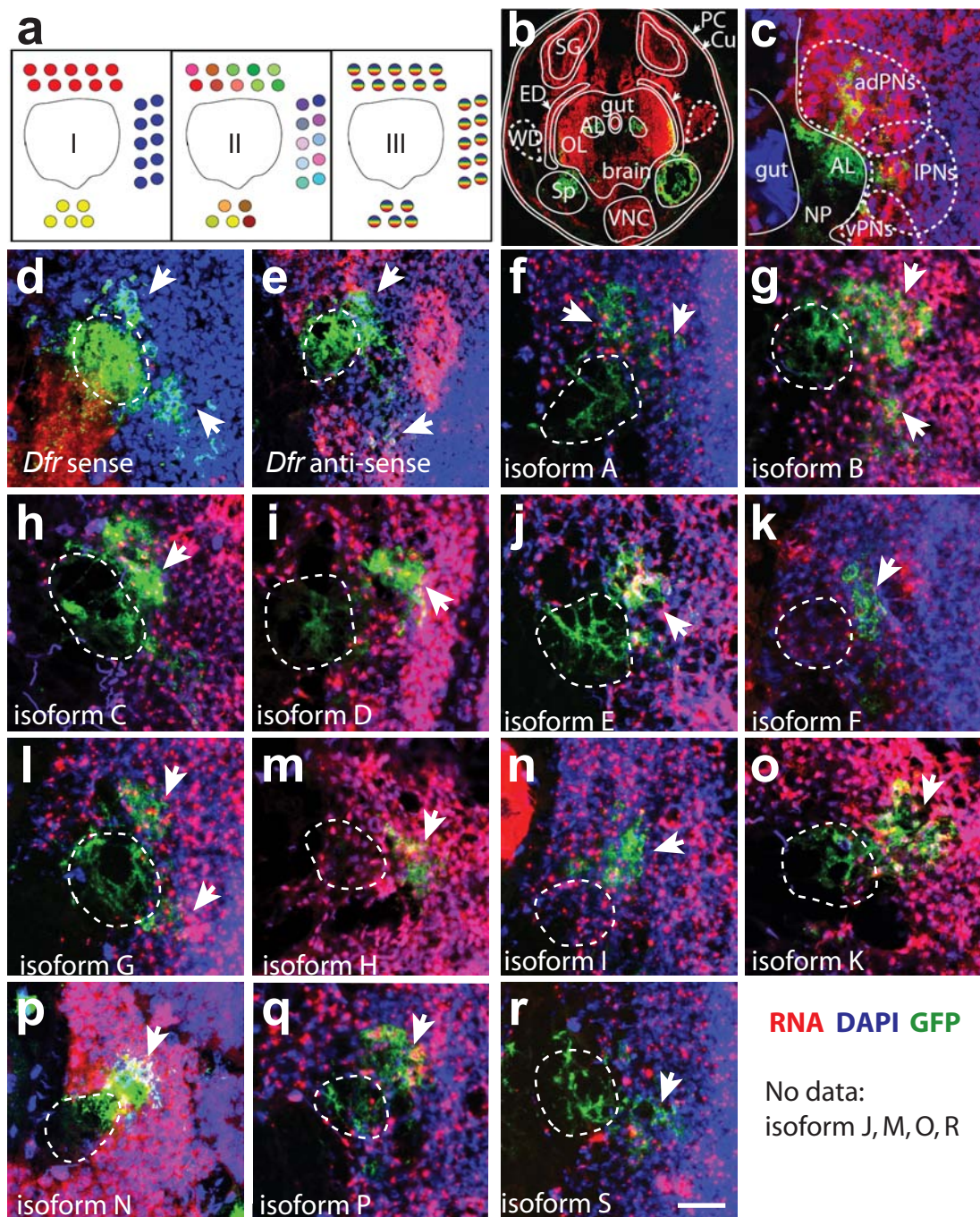

Supplement: Additional file 3 — Additional lola isoform in situ in the Drosophila brain. Supplemental Figure S2 showing controls and verification for our in situ technique and additional lola isoform hybridization results. [file 1749-8104-2-14-S3.pdf]

# Figure S4

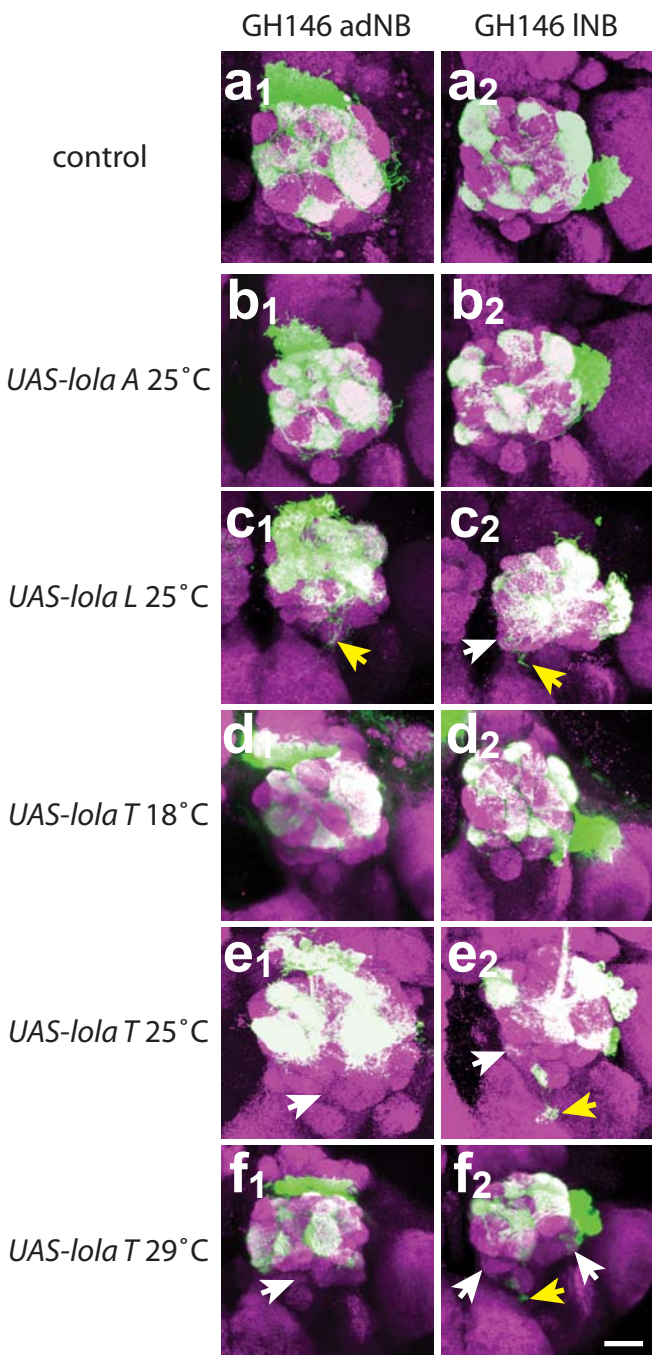

Supplement: Additional file 5 — Effects of UAS-lola overexpression on adPN and lPN dendrites and axons. Supplemental Figure S4 showing adPN and lPN phenotypes in MARCM clones in a wild-type background expressing UAS-lola A, UAS-lola L and UAS-lola T. [file 1749-8104-2-14-S5.pdf]
