# Supplementary material for: Lola regulates Drosophila olfactory projection neuron identity and targeting specificity
Source: Neural Dev. 2007 Jul 16;2:14. doi: 10.1186/1749-8104-2-14 (PMC1947980; doi:10.1186/1749-8104-2-14)
Supplement: Additional file 2 — Supplemental Tables S1-S6. Supplemental tables and legends referenced in the main text. This includes a semi-quantitative analysis of axon targeting defects in lola-/- and lolaORE5D20-/-, a complete summary of DL1 dendrite and axon phenotypes by allele as percentage affected, a complete summary of vPN dendritic phenotypes by allele as percentage targeted correctly, clonal frequencies for MARCM experiments and primer sequences for in situ hybridization probes. [file 1749-8104-2-14-S2.doc]

Table S1. DL1 dendrite phenotypes (by allele as % affected)

| Genotype | WT | Medium | Severe | N |
| --- | --- | --- | --- | --- |
| control | 100.0% | 0.0% | 0.0% | 46 |
| *lola*^ORE76^ (null) | 23.0% | 54.0% | 23.0% | 26 |
| *UAS-lola A*, null^-/-^ | 50.0% | 25.0% | 25.0% | 8 |
| *UAS-lola L*, null^-/-^ | 0.0% | 100.0% | 0.0% | 7 |
| *UAS-lola T*, null^-/-^ | 0.0% | 63.6% | 36.4% | 11 |
| *UAS-lola A* | 100.0% | 0.0% | 0.0% | 35 |
| *UAS-lola L* (#3) | 66.7% | 33.3% | 0.0% | 15 |
| *UAS-lola L* (#5) | 70.4% | 14.8% | 14.8% | 54 |
| *UAS-lola L* (#9) | 0.0% | 96.0% | 4.0% | 25 |
| *UAS-lola L* | 51.1% | 39.4% | 9.6% | 94 |
| *UAS-lola T* (25) III | 0.0% | 55.0% | 45.0% | 20 |
| *UAS-lola T* (25) X | 65.8% | 31.6% | 2.6% | 76 |
| *UAS-lola T* (25) | 51.4% | 38.1% | 10.5% | 105 |
| *UAS-lola T* (18) | 100.0% | 0.0% | 0.0% | 39 |
| *UAS-lola T* (29) | 38.3% | 53.2% | 8.5% | 47 |

Table S1. DL1 dendritic phenotypic classification as % affected for all alleles examined. A medium phenotype indicates some innervation of DL1 in addition to ectopic dendritic extensions into the AL that often innervate glomeruli anterior to DL1 in the AL. A severe phenotype indicates an entire lack of targeting to DL1.

**Table S2. Semi-quantitative analysis of axon targeting defects in *lola^-/-^* and *lola^ORE5D2-/-^***

|  |  | ectopic branches | lack of innervation | misrouting | SOG | "N" |
| --- | --- | --- | --- | --- | --- | --- |
|  | GH146 DL1 | 0% | 0% | 0% | 0% | 46 |
| control | GH146 adNB | 0% | 0% | 0% | 0% | 22 |
|  | GH146 lNB | 0% | 0% | 0% | 0% | 20 |
|  | GH146 vNB | 0% | 0% | 0% | 0% | 34 |
|  | GH146 DL1 | 20% | 0% | 18% | 0% | 22 |
| hypomorph | GH146 adNB | 25% | 4% | 4% | 0% | 23 |
| (ore5D2) | GH146 lNB | 33% | 0% | 9% | 0% | 11 |
|  | GH146 vNB | 45% | 0% | 41% | 47% | 17 |
|  | GH146 DL1 | 26% | 15% | 0% | 0% | 26 |
| lola^-/-^ | GH146 adNB | 28% | 6% | 3% | 12% | 73 |
| (ore76) | GH146 lNB | 63% | 14% | 7% | 0% | 44 |
|  | GH146 vNB | 79% | 9% | 34% | 9% | 35 |

Table S2. *lola*^-/-^ and *lola^ore5D2^*^-/-^ specific axon targeting defects. Both *lola* alleles show a marked increase in ectopic branching from both the MB and LH regions. *lola*^-/-^ clones have an increased rate of loss of MB or LH innervation where axons fail to extend processes into normal target regions. Both alleles show misrouting phenotypes, most markedly in vNB clones where a single axon often defasiculates and projects directly to the medial side of the LH. Often *lola*^-/-^ axons defasiculate posterior to the AL from the main axon bundle and project to the SOG.

Table S3. DL1 axon phenotypes (by allele as % affected)

| Genotype | WT | Mild | Medium | Severe | N |
| --- | --- | --- | --- | --- | --- |
| control | 100% | 0% | 0% | 0% | 46 |
| lolaORE76 (null) | 35% | 15% | 31% | 12% | 26 |
| UAS-lola A, null-/- | 38% | 25% | 13% | 25% | 8 |
| UAS-lola L, null-/- | 0% | 0% | 14% | 86% | 7 |
| UAS-lola T, null-/- | 9% | 36% | 27% | 27% | 11 |
| UAS-lola A | 74% | 23% | 3% | 0% | 35 |
| UAS-lola L (#3) | 13% | 33% | 53% | 0% | 15 |
| UAS-lola L (#5) | 50% | 31% | 19% | 0% | 54 |
| UAS-lola L (#9) | 0% | 52% | 32% | 16% | 25 |
| UAS-lola L | 31% | 37% | 28% | 4% | 94 |
| UAS-lola T (25) III | 0% | 15% | 80% | 5% | 20 |
| UAS-lola T (25) X | 25% | 45% | 29% | 1% | 76 |
| UAS-lola T (25) | 20% | 39% | 40% | 2% | 96 |
| UAS-lola T (18) | 69% | 31% | 0% | 0% | 39 |
| UAS-lola T (29) | 13% | 32% | 55% | 0% | 47 |

Table S3. DL1 axon phenotypes as % affected for all alleles examined. A mild phenotype indicates axons that target to the MB and LH, but have significant extensions outside of those regions. A medium phenotype indicates axons that ultimately target to the MB and LH, but either failure to innervate on of these regions, follow and incorrect trajectory to these regions or bifurcate. A severe phenotype indicates axons that do not project to the MB and LH and either target the SOG or another brain region.

Table S4. vPN dendritic phenotypes (by allele, % that target correctly)

| Genotype | DA1 | VA1lm | SOG | "N" |
| --- | --- | --- | --- | --- |
| control | 82.4% | 82.4% | 0.0% | 34 |
| *lola*^ORE76^ (null) | 2.9% | 28.6% | 25.7% | 35 |
| *lola*^ORE5D2^ (hypo) | 0.0% | 0.0% | 0.0% | 17 |
| *lola*^ORE119^ (*lola L^-/-^*) | 94.7% | 94.7% | 0.0% | 19 |
| *UAS-lola A*, null^-/-^ | 0.0% | 83.3% | 83.3% | 12 |
| *UAS-lola L*, null^-/-^ | 14.3% | 28.6% | 14.3% | 7 |
| *UAS-lola T*, null^-/-^ | 0.0% | 80.0% | 80.0% | 5 |
| *UAS-lola A* | 78.7% | 83.0% | 4.3% | 47 |
| *UAS-lola L* (#3) | 83.3% | 83.3% | 0.0% | 12 |
| *UAS-lola L* (#5) | 88.0% | 88.0% | 0.0% | 25 |
| *UAS-lola L* (#9) | 90.0% | 90.0% | 0.0% | 20 |
| *UAS-lola L* | 87.7% | 87.7% | 0.0% | 57 |
| *UAS-lola T* (25) III | 0.0% | 0.0% | 0.0% | 7 |
| *UAS-lola T* (25) X | 36.5% | 36.5% | 0.0% | 63 |
| *UAS-lola T* (25) | 32.9% | 32.9% | 0.0% | 70 |
| *UAS-lola T* (18) | 95.0% | 95.0% | 0.0% | 20 |
| *UAS-lola T* (29) | 45.0% | 45.0% | 0.0% | 40 |

Table S4. vPN dendritic phenotypes of all alleles examine presented as % of clones that innervate the listed glomerulus or brain region. The most prominent *lola* mutant phenotype is a loss of DA1 innervation in vNB clones. Other manipulations also affect VA1lm innervation and cause ectopic dendritic extensions to the SOG. Most vNB clones do not display a consistent loss of cell bodies, but a loss in specific dendritic innervation.

Table S5. Clonal frequencies for MARCM experiments

| Genotype | DL1 | DNB | LNB | VNB | non-PN |
| --- | --- | --- | --- | --- | --- |
| control | 37.7% | 18.0% | 16.4% | 27.9% | 0.0% |
| *lola*^ORE76^ (null) | 11.4% | 31.9% | 19.2% | 15.3% | 22.3% |
| *lola*^ORE5D2^ (hypo) | 29.3% | 30.7% | 14.7% | 22.7% | 2.7% |
| *lola*^ORE119^ (*lola L^-/-^*) | 32.3% | 14.5% | 21.0% | 30.6% | 1.6% |
| *UAS-lola A*, null^-/-^ | 10.3% | 43.6% | 15.4% | 15.4% | 15.4% |
| *UAS-lola L*, null^-/-^ | 25.0% | 32.1% | 10.7% | 25.0% | 7.1% |
| *UAS-lola T*, null^-/-^ | 28.2% | 20.5% | 25.6% | 12.8% | 12.8% |
| *UAS-lola A* | 31.8% | 30.6% | 10.0% | 27.6% | 0.0% |
| *UAS-lola L* (#3) | 35.7% | 26.2% | 9.5% | 28.6% | 0.0% |
| *UAS-lola L* (#5) | 50.9% | 21.1% | 6.1% | 21.9% | 0.0% |
| *UAS-lola L* (#9) | 36.2% | 26.1% | 8.7% | 29.0% | 0.0% |
| *UAS-lola L* | 43.6% | 23.6% | 7.6% | 25.3% | 0.0% |
| *UAS-lola T* (25) III | 42.6% | 25.5% | 17.0% | 14.9% | 0.0% |
| *UAS-lola T* (25) X | 31.3% | 28.0% | 14.8% | 25.9% | 0.0% |
| *UAS-lola T* (25) | 33.1% | 27.6% | 15.2% | 24.1% | 0.0% |
| *UAS-lola T* (18) | 44.8% | 24.1% | 8.0% | 23.0% | 0.0% |
| *UAS-lola T* (29) | 30.8% | 30.2% | 13.8% | 25.2% | 0.0% |

Table S5. Clonal frequencies in MARCM experiments presented as overall % of clone type by allele. *lola*^-/-^ cells show a marked reduction in labeling of adSC clones, and a marked increase in the labeling of ectopic clone types, in particular LN labeling. Overexpression results in changes in lNB clonal frequency.

**Table S6. Primer sequences for *in situ* hybridization probes.**

| In-situ Probe Name | 5' (left) primer | 3' (right) primer |
| --- | --- | --- |
| *acj6* | GGTTGGGGGTTTAGCTCTCT | GCCTGTCTGGGCGACTATTA |
| *drifter* | AGCATAGGGACCCTGGTCTT | AGATGCGACATCTCCTGCTT |
| *lola* common region | GCCCACGACAACTCAAACTCG | TCGGAGCCCACTGATGACAC |
| *lola* isoform A | ATCCCCAAGGTGAGTGTTTG | CTTCTTCAACGTTTTAGAGC |
| *lola* isoform B | CAATGGTTGTGCCCAAAATCACC | CGGGTGTTTTACATCGGTTACAC |
| *lola* isoform C | GTTTTACTATGACAGCGAAATGCC | GTATTGTGCTTCTTGACCTTGC |
| *lola* isoform D | GATGGCAGCAGCGATGACTATC | GTTCATCCTCATCGTCATCTTCG |
| *lola* isoform E | AACCAGAACCTCAACCATCA | GCTATTGCCGCTACTGTTGT |
| *lola* isoform F | CGGCAATTGCACAGATGACAAC | GTTCATCAGGTGCTGCACCTTG |
| *lola* isoform G | CGACCGAACCGTATCCGTATC | AAGCTCCAAGGACGCACACGC |
| *lola* isoform H | CGTACTAACCTGGTATCAGCAC | TATGCGGTGGCCTCCCATTTG |
| *lola* isoform I | CAAGCAGCTATGTCTCCAACTC | TCCTCTTGCTGTATTGCGTGTG |
| *lola* isoform J | TCCTGGACGATATCCGTGAAATC | CATTAGAAGTGGTCTCTGGTCTC |
| *lola* isoform K | TACCGGCAACCACAACAATTGC | CATATGCTCAGTAATCTCCGAGG |
| *lola* isoform L | GCTGGCGTTGGCATAACCAC | TCCGTTTTCCCGAGAGCTTG |
| *lola* isoform M | CAGTCTTACATATTGTTTTGTTGC | CTGAAGGTTAGTTGTTTGTCGG |
| *lola* isoform N | GCCACCATTACGACGATTTC | ATCGTTCTGGAACACCAAGG |
| *lola* isoform O | GATAACGACGACAGCAACGA | AGGTGTTTCCGCATCTATCG |
| *lola* isoform P | CTATCCGATCCTTGGCAGTCTTC | TTGATCTCACAAAGAGCGGCAGC |
| *lola* isoform Q | GAGACGGTGCCCTATTCGATTG | CATCGTGCTGGTCAACCTTCATG |
| *lola* isoform R | GAACCAGCTCCTGTGCCAATG | GACAGGACAAGACATTCGCTTG |
| *lola* isoform S | GTTCTTCGGCAGATCTGCCAAG | GACTGACATGAGTTGGATTTTCC |
| *lola* isoform T | TAGATCCCTCGACCATTTCG | GCATGATAGCGACCCTCTTC |
