# Supplementary material for: Lola regulates Drosophila olfactory projection neuron identity and targeting specificity
Source: Neural Dev. 2007 Jul 16;2:14. doi: 10.1186/1749-8104-2-14 (PMC1947980; doi:10.1186/1749-8104-2-14)
Supplement: Additional file 4 — Additional lola isoform RT-PCR analysis in the Drosophila brain. Supplemental Figure S3 showing addition LCM RT-PCR experiments comparing lola isoform expression at different developmental time points and in different brain tissues. [file 1749-8104-2-14-S4.pdf]

Figure S3

a **Developmental timecourse of *lola* isoform expression in AL**

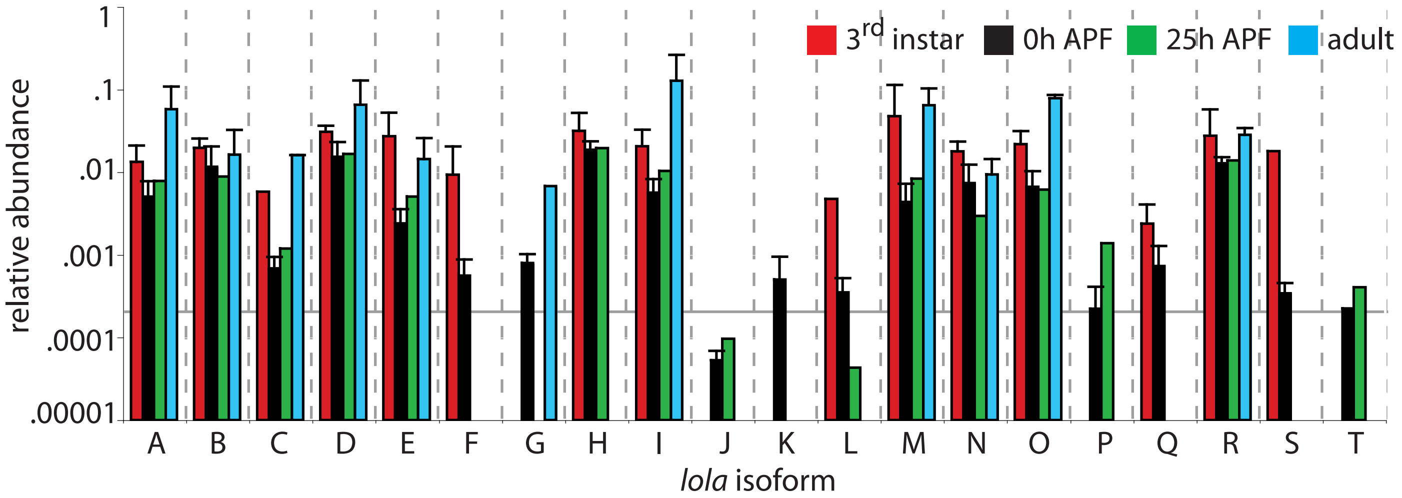

b **Developmental timecourse of *lola* isoform expression in optic lobe**

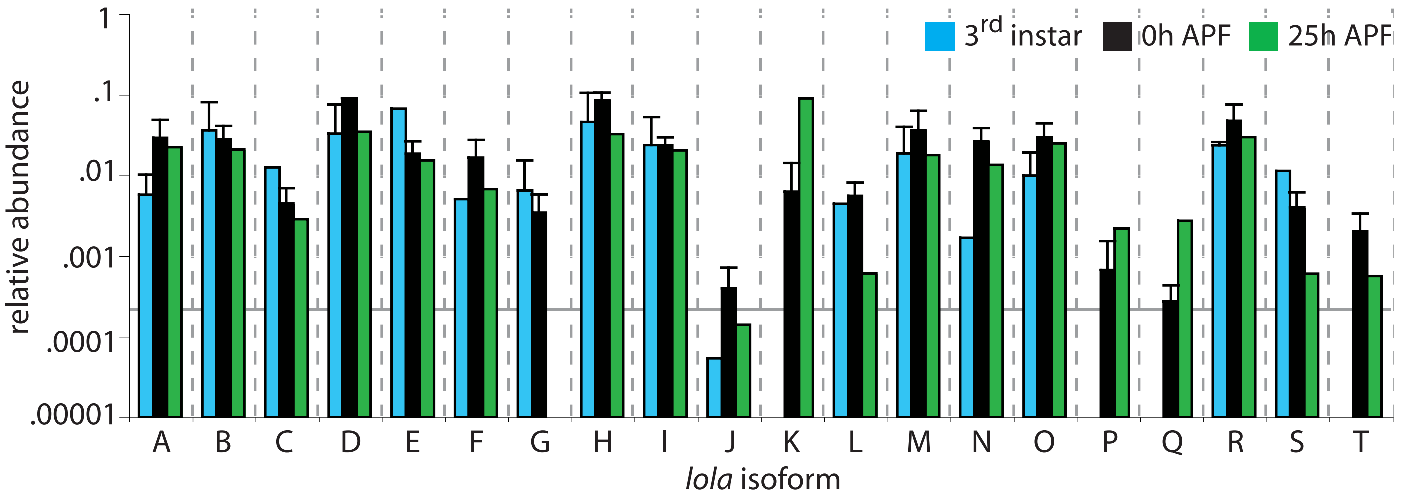

c **Comparison of *lola* isoform expression at 0h APF between brain subregions**

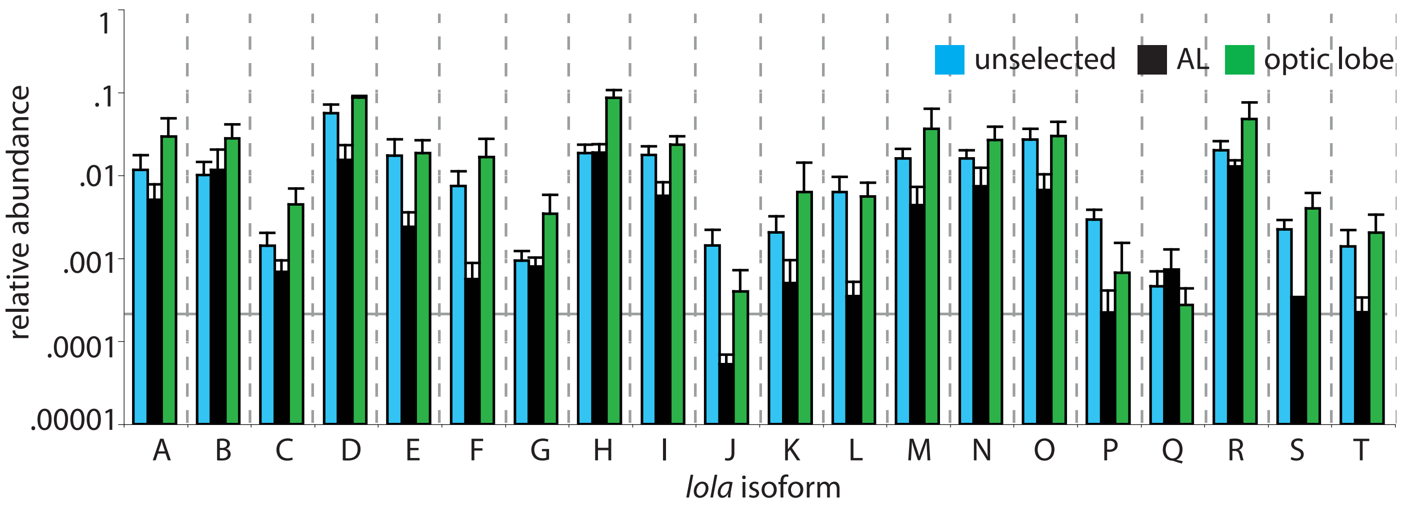

Spletter, et al.
