# Supplementary material for: Lola regulates Drosophila olfactory projection neuron identity and targeting specificity
Source: Neural Dev. 2007 Jul 16;2:14. doi: 10.1186/1749-8104-2-14 (PMC1947980; doi:10.1186/1749-8104-2-14)
Supplement: Additional file 6 — adNB and lNB phenotypes in lola-/-, UAS-lola MARCM clones. Supplemental Figure S5 showing adPN and lPN phenotypes in lola-/- clones expressing UAS-lola A, UAS-lola L and UAS-lola T. [file 1749-8104-2-14-S6.pdf]

Figure S5

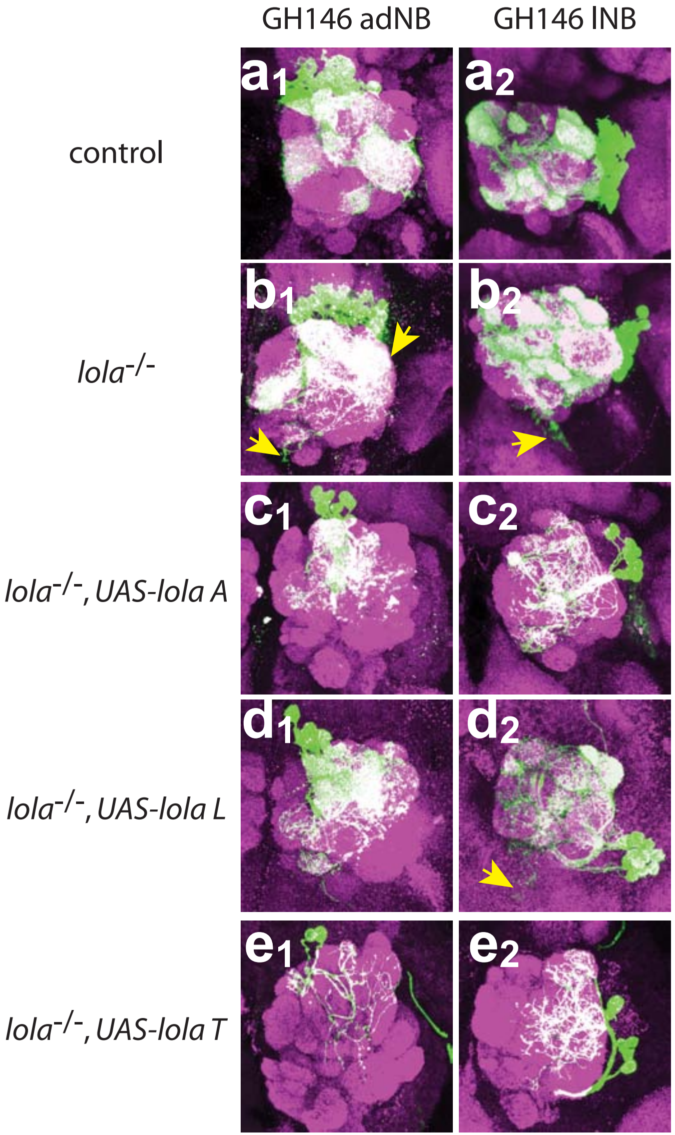

Spletter, et al.
