# Supplementary material for: Lola regulates Drosophila olfactory projection neuron identity and targeting specificity
Source: Neural Dev. 2007 Jul 16;2:14. doi: 10.1186/1749-8104-2-14 (PMC1947980; doi:10.1186/1749-8104-2-14)
Supplement: Additional file 7 — Protocol for RNA in situ hybridization in fly larval/pupal/adult tissues. Our detailed protocol for performing RNA in situ hybridization on Drosophila at later developmental time points. [file 1749-8104-2-14-S7.doc]

Supplemental Protocol for RNA *in situ* hybridization in fly larval/pupal/adult tissues

Many protocols for in-situ hybridization in *Drosophila* exist, but most focus on embryonic stages. We have adapted several protocols for non-radioactive *in situ* hybridization [1-3] and integrated tyramide signal-based amplification [4] for use in *Drosophila* larval, pupal and adult tissues. Our protocol consists of four main stages: (1) probe generation, (2) tissue preparation, (3) hybridization and (4) probe detection. This protocol details steps and suggestions for each stage.

**I. Probe Generation**

***Overall goal:***

Generate sense and antisense probes to your specific gene of interest. Probes are PCR amplified and cloned into the Topo vector for verification. Subsequently probes are in-vitro transcribed and labeled with digoxigenin (DIG) or biotin for in-situ detection.

***Necessary Reagents*** (†denotes a recipe is given at the end of the protocol)***:***

•Topo pCR2.1 (Dual Promoter vector, Invitrogen #K460001)

•probe-specific primers

•RT-PCR reagents

•phenol-chloroform

•chloroform (RNase free)

•3 M NaAc (pH 5.2) (RNase free)

•EtOH (100% & 75%) (RNase free)

•Roche RNA in vitro transcription and labeling kit (DIG-RNA labeling kit #11175025910 or Biotin RNA labeling mix #11685597910)

•0.5 M EDTA pH 8 (RNase free)

•7.5 M LiCl (RNase free)

•0.3-0.5 M NaOH

•RNase inhibitor (Roche #3335399001 or Invitrogen #15518012)

•Hybridization buffer† (RNase free)

***Probe Design and Cloning***

1. Design primers to amplify probe region

*Design primers for a probe sequence around 400-600 bp in length (we have been successful with a 220 bp probe but not with anything shorter, and most reliable probes are ~500bp). Be sure to design the probe to unique regions of your sequence (BLAST your probe ahead of time to make sure it only recognizes your gene). Probes across exons are more effective in identifying mature RNA as they do not recognize the DNA sequence or pre-mRNA.*

2. RT-PCR amplify the probe using standard PCR protocols

3. Clone the PCR product directly into Topo pCR2.1

4. Sequence verify Topo insertions

*We have found that probe PCR can be dirty and amplify more than the probe of interest. Cloning into Topo is more work intensive, but allows one to amplify the plasmid faithfully in bacteria to have sufficient pure starting material for probe synthesis, and to verify the identity of the probe and pick probes without mutations. Also, cloning into the dual promoter Topo vector allows generation of sense and antisense probes from the same starting material.*

***In-vitro Transcription and probe labeling***

1. identify restriction sites for sense and antisense probes

*To get a specific probe that does not contain the topo vector sequence, the vector needs to be restriction digested prior to probe transcription and labeling. We typically choose a restriction site in the polylinker that is not found in the probe itself so amplification will proceed through the probe and stop at the point of the restriction cut. For example, we would determine based on sequence orientation if transcription using the SP6 promoter would produce the sense or antisense probe, and then pick a unique restriction site in the T7 linker arm to cut.*

2. Cut ~20 µL (10 ng) of a miniprep with a single enzyme to linearize.

*We typically incubate for 2 hours at 37°C to ensure the digestion goes to completion. The digestion volume should be at least 4X the volume of DNA used (ie if you start with 20 µL, use a digest volume of 80 µL). This prevents crowding and ensures the DNA is accessible so the restriction can proceed to completion.*

3. Dilute the reaction to 100-200 µL.

4. Phenol-chloroform extract the digested DNA

- 1. ***from this point forward use RNase-free techniques and solutions***
  2. add phenol:chloroform (1:1) to the diluted RE reaction 1:1
  3. shake tube to mix well
  4. spin for 2-3 minutes to separate phases at 13,000 rpm at 4°C
  5. transfer top clear/aqueous liquid phase to a new RNase free tube
  6. add chloroform 1:1
  7. shake tube to mix well
  8. spin for 2-3 minutes to separate phases
  9. remove top clear liquid phase containing DNA
  10. add 3 M NaAc pH 5.2 1:10 of liquid phase
  11. add 2.5X volume of cold 100% EtOH
  12. precipitate DNA at -20°C overnight or at -80°C for ~30 min.
  13. spin for 2-3 minutes at 13,000 rpm at 4°C
  14. remove supernatant from pellet
  15. wash in 75% EtOH
  16. rinse in 100% EtOH and dry briefly on the bench
  17. resuspend in water and take OD
  18. run a gel to verify that the plasmid was fully linearized

5. In-vitro transcription

*Follow the manufacturers protocol as summarized below for the Roche RNA in vitro transcription and labeling kit (can do a ½ reaction and works well). Probes can be labeled with either DIG or biotin. Both can be adequately detected. We find that DIG labeled probes last longer and give less background.*

1. add water, buffer, DNA, labeling mix, RNase inhibitor, SP6 or T7 polymerase
2. incubate 2 hours at 37°C
3. add 2 µL DNase/RNase free and incubate at 37°C for 15 minutes
4. add 2 µL of 0.5 M EDTA pH 8
5. add 1.6 µL 7.5M LiCl
6. add 2.5X volume cold 100% EtOH to precipitate
7. precipitate DNA at -20°C overnight or at -80°C for ~30 min.
8. spin and remove supernatant from pellet
9. wash in 75% EtOH
10. rinse quickly in 100% EtOH and dry quickly on the bench
11. resuspend in 30-50 µL water and OD
12. run 3 µL on a gel and verify probe integrity

*We have found this important to verify that in-vitro transcription worked well and that the sample was not contaminated with RNase. Reactions that give improperly size products or weak products or display laddering should be redone before labeling. Before running the verification gel, wash the gel box with 0.3-0.5M NaOH for 30 min. and rinse with Millipore water. Samples can be run in standard TBE/TAE agarose gels by loading samples with 1 µL of RNase inhibitor to prevent degradation.*

1. after OD adjust volume to 10 ng/µL (or whatever concentration you prefer for your probe stock) in hybridization buffer

*Probes should be diluted in the hybridization buffer that will be used for subsequent in-situ hybridization. We provide two recipes for hybridization buffer at the end of the protocol. In our hands, although both hybridization buffers work, the dextran-sulfate containing buffer (Type II) preserves morphology of sections better and has less background than the Type I buffer.*

1. aliquot in 20-50µL aliquots and freeze at -80°C
2. probes can be stored for 6-8 months

**II. Tissue Preparation**

***Overall Goal:***

Prepare samples for sectioning, section samples and collect serial sections at the time-point(s) of interest without mRNA degradation. For this paper we performed all in-situ experiments at 0h APF. We have also been successful at sectioning various time-points from larva to adult and expect this protocol to work on almost any post-embryonic fly tissue.

***Necessary Reagents:***

•Tissue-Tek Intermediate Cryomold (Tissue-Tek #5466)

•Tissue-Tek O.C.T. Compound (Tissue-Tek #4583)

•RNase AWAY (Invitrogen #10328-011)

•Superfrost Plus microslides (VWR International #48311-703)

•dry ice

***Collect samples:***

1. Collect flies at the timepoint of interest. (We collected white pre-pupa and considered them to be 0h APF.)

2. Line-up samples along one edge of a Tissue-Tek Intermediate Cryomold.

*If you are collecting larvae, at this point you may want to place them on ice so they stop moving so you can align them. For both larvae and pupa, attempt to align the brain (visible as a more transparent region of the animal near the mouth), and not just the anterior-most extremity of the animals. This is important while sectioning to maximize the number of samples of the region of interest per slide. For adults, whole heads can be aligned and sectioned successfully. For pupae and adult heads, adding a thin stripe of O.C.T. along the bottom of the cryomold can make alignment easier as you can “stick” your samples in place.*

3. Cover samples with Tissue-Tek O.C.T. medium, taking care to not create bubbles or disrupt the alignment of samples.

4. Place cryomolds containing samples and O.C.T. on dry ice and let them freeze completely

5. Place and store frozen samples at -80°C for up to 1 month

***Section samples:***

1. Section samples in 10-20 µM thick sections on a cryostat

*Remember to be RNase conscious during this procedure. Try not to introduce RNase to the samples and limit the activity of native RNases. Use only new tools that have been washed with RNase Zap or RNase AWAY or a similar product. Section as efficiently as possible and be aware that the longer you keep samples and sections at warmer temperatures, the more effect native RNases will have on sample integrity. We find that sectioning with a fixed blade instead of disposable razor blades provides better section morphology and reproducibility. Before use, wash the blade with RNase AWAY and rinse with RNase-free water. Be sure your blade is sufficiently sharp to cut well. With adult and pupal samples you will be cutting through cuticle/pupal case, and a dull blade that does not cut cleanly will only destroy the soft tissues of interest. Remember to pay attention to the orientation of your block so you know which direction is ventral versus dorsal in the sections.*

2. Mount sections on Superfrost Plus slides (the charge helps keep the sections on the glass during subsequent wash steps)

*We verify the presence of GFP signal for our cells of interest and directly store slides in a slide box on dry ice without additional drying of the sections. For doing sense versus anti-sense comparisons, it is often easier if sections are split between two slides and mounted every-other section (ie one section on slide 1, the next on slide 2, then the next on slide 1 and so forth), so that the same exact animal and relative position can be compared between two different probes.*

3. Store slides in a box on dry ice while sectioning additional samples.

4. Slides can be stored at -80°C overnight before proceeding to hybridization.

**III. Hybridization**

***Overall Goal:***

This section of the protocol includes all steps relevant to tissue fixation, tissue preparation, hybridization and post-hybridization washes. This part of the protocol is RNase free for all reagents and techniques! We find that the maximum number of slides that can be adequately processed per person at the same time is 20 (due to time sensitivity of certain steps during probe detection). This point to the end of the protocol will take roughly 3 days to complete.

***Necessary Reagents:***

Day 1

•4% paraformaldehyde (PFA)† (RNase free)

•1x PBS† (RNase free)

•SPI plastic slide mailers (www.2spi.com, #01253A)

•0.3-0.5M NaOH

•0.1 % PBS-triton† (RNase free)

•acetic anhydride (RNase free)

•TEA† (RNase free)

•humidified slide chamber

•hybridization buffer† (RNase free)

•RNase-free disposable coverslips, 24x60mm (Research Products International Corp. #247457)

•hybridization oven preheated to 55-60°C

Day 2

•formamide wash buffer† (prewarmed to 55-60°C)

•2x SSC† (RT and prewarmed to 55-60°C)

•0.2x SSC† (prewarmed to 55-60°C)

•plastic dropper (Samco Transfer Pipettes, #202)

•PBS-triton†

•H_2_O_2_

•methanol

•TBS-T†

Day 1:

***Fixation*:**

1. Fix slides for ~15 min. in 4% PFA in 1X PBS at RT.

*We fix in slide mailers from SPI supplies that hold 5 slides in ~15 mL of fix each. Fix and washes can easily be removed without handling the slides (handling increases the risk of RNase contamination) and the holder can easily be treated with 0.3-0.5M NaOH to remove all RNase prior to fixation. We fix slides directly upon removal from -80°C without waiting for slides to thaw or dry. The time of fixation ranges from 10-20 minutes depending on thickness of section and age of the tissue. If you notice tissue is brittle and “snaps” during subsequent steps, fix for less time. Formaldehyde can be used as a substitute fixative, but we have poor results fixing fly tissue with Zn-formalin.*

2. Wash slides 3 times for 5 min. each in RNase free 1x PBS-triton (0.1% triton) or TE (Tris-HCl, pH 8.0) on a shaker at low speed.

***Acetylation:***

3. Add 125 µL of acetic anhydride to 50 mL of TEA and immediately incubate on slides for 10 minutes at room temperature.

*This acetylation step increases specific signal intensity and helps control background. Although acetylation is recommended to be carried-out in a glass container, we see little difference between slides acetylated in plastic SPI containers or glass stain-boats.*

4. Wash slides 3 times for 5 min. each in RNase free PBS-triton on a shaker at low speed.

***Prehybridization:***

5. Prepare a humidified chamber for hybridization.

*These can be purchased or homemade by placing pipettes on the bottom of square plastic dishes (with covers) and placing wetted paper towel or kim-wipes between the pipettes.*

6. Transfer slides to humidified chamber (place on pipettes above wetted tissue).

7. Prehybridize by adding 500-700 µL of hybridization buffer per slide and incubating for 2 hours (minimum 30-60 minutes) at room temperature.

8. During pre-hybridization, dilute the desired amount of riboprobe in 100 µL of hybridization buffer.

*The correct concentration of riboprobe varies between probes. For most probes, the ideal concentration falls somewhere between 10-30 ng per slide. This gives the lowest background and highest specific signal. A medium-high signal and background should be expected from 40-70 ng and a high signal for 80-150 ng. We recommend starting with at least two concentrations (one low, one high or mid) and titre the concentration of probe based on those results. Every probe will be different. There is also often a thresh-holding effect where a probe may give good signal at low concentrations, but may stain all tissues non-specifically at high concentrations. In our hands, probes also often non-specifically bind to the cuticle, gut, mouth-hooks and fat bodies sometimes even at low concentrations.*

9. Boil the diluted probe at 80°C for 5 minutes in the PCR machine to denature.

10. Remove the probe immediately and place on ice.

***Hybridization:***

11. Dump pre-hybridization buffer off of slides and add the full 100 µL of riboprobe.

12. Cover each slide with a plastic RNase-free coverslip (Research Products International Corp. #247457) to help prevent evaporation.

13. Place cover-slipped slides back in the humidity chamber.

14. Wrap the humidity chamber in foil to help seal and prevent evaporation of all liquid in the chamber.

15. Place the chamber in an oven pre-heated to 55-60°C for 16-20 hours.

*If you are using Type I hybridization solution incubate at 60°C. If you are using Type II hybridization solution with dextran sulfate incubate at 55°C. We find that specific signal requires incubation for a minimum of 12 hours, and the best signal is obtained around 18 hours.*

Day 2

***Post-hybridization washes:***

1. Pre-warm all wash buffers to 55-60°C before using (same temperature you used for hybridization), and thaw and add formamide to formamide wash buffer.

2. Remove hybridization chamber from oven.

3. Using a plastic dropper, add 2x SSC to a slide to “float” plastic coverslip for easy removal (coverslip can be tipped off of the slide). Rinse with a dropperful of 2x SSC and place in a glass wash boat filled with warm 2x SSC. Repeat until the coverslips have been removed and all slides are in the glass wash boat.

4. Rinse slides in warm 2x SSC.

5. Replace rinse with warm formamide wash buffer.

6. Place glass wash boat back in the oven on a shaker at low speed. Wash a total of 3 times for 30 minutes on a shaker in formamide wash buffer at the hybridization temperature.

7. Wash one time in 0.2x SSC for 30 min. in the oven on a shaker at the hybridization temperature.

8. Remove the wash boat from the oven and wash slides in 1x PBS-triton for 20 minutes at on shaker at RT to cool slides and tissue to room temperature.

***Quench native HRP signal:***

9. To quench native HRP, incubate in 0.3% H_2_O_2_ in methanol (300 µL hydrogen peroxide: 100 mL MeOH) without shaking for 20 min. at room temperature.

10. Wash in ddH_2_O for 10 min.

11. Rinse in TBS-T.

**IV. Probe detection**

***Overall goal:***

This part of the protocol covers fluorescent signal detection. It is not necessary to be RNase free post-hybridization, so all block and wash buffers may be made without following RNase-free technique. All fluorescence antibody steps should be carried out in a humidified chamber to prevent evaporation and tissue drying. Do not let the tissue dry at any point in this protocol or staining will be poor with high background and loss of specificity. The protocol varies slightly for RNA probes labeled with DIG or with biotin. Additionally, we often find it necessary for genes with low levels of expression to use a tyramide-based signal amplification system to see the *in-situ* probe signal. We discuss detection with and without signal amplification.

***Necessary Reagents:***

•serum (normal donkey serum or normal goat serum [Lampire Biological Laboratories #7332100 or #7332500]) or blocking reagent (Roche #1096176)

•rabbit anti-DIG-HRP (DAKO #P5104; use 1:200-1:2000)

•sheep anti-DIG-POD-HRP (Roche Scientific #11593700; use 1:500-1:100)

•rabbit anti-GFP (Molecular Probes #A-6455) or chicken anti-GFP (Aves Labs, Inc. #GFP-1020)

•Cy3-tyramide (Perkin-Elmer #SAT704A)

•Alexa 488 anti-rabbit (Molecular Probes #A11034) or FITC anti-chicken (Jackson ImmunoResearch #703-095-155)

•DAPI (1 mg/mL, use 1:1000)

•Biomedia Mounting Medium Gel/Mount (Biomedia, #M01)

•Glass Coverslips (VWR International No. 1 22x50mm #48393-059)

•DAKO TSA kit (#K0620) or Perkin-Elmer TSA kit (NEL700A) or Perkin-Elmer Cy3-TSA kit (NEL704A)

•Cy3-Streptavidin (Jackson ImmunoResearch #016-160-084)

Day 2 (continued) & Day 3 (if signal amplification is done overnight at 4°C)

***DIG labeled-probe detection:***

1. Block slides at RT for 30-60 minutes in 10% serum in TBS-T

*Serum should be picked according to the species of the secondary antibody, i.e. for a goat secondary use NGS, for a donkey secondary use NDS. Slides can also be blocked in TBS-T plus blocking buffer.*

2. Add primary antibodies in 5% serum in TBS-T for 1-2 hours at RT

*Remember to include anti-DIG-POD-HRP for DIG-labeled probe detection as well as any additional primary antibodies (i.e. anti-GFP). There are two main anti-DIG-POD-HRP antibodies available, a rabbit anti-DIG-POD-HRP (used 1:500) and a sheep anti-DIG-POD-HRP (used 1:500-1000). Both work but sheep has a higher background signal in our hands. The DIG antibodies are fairly specific and give good signal after 2 hours at room temperature. However, they can be incubated at 4°C overnight without negative effects. Primary antibodies for other detection should also be added. For example, if your cells of interest are labeled with GFP, add a primary anti-GFP. We find that both rabbit anti-GFP and chicken anti-GFP work well (we also tried rat-anti-CD8 but failed to detect any CD8:GFP signal with that antibody in in-situs). We find that GFP signal itself is mostly destroyed or very weak after previous in-situ hybridization steps, so immuno-detection is necessary.*

3. Wash slides 3 times for 15 min. each in TBS-T.

4a. (no signal amplification) Add Cy3-Tyramide for 10 min. at RT

*Cy3-Tyramide provides strong signal detection for HRP. Concentrations must be tested to find the correct level of signal. We suggest 1:70, 1:150 or 1:250. Signal strength can also be modulated by changing the length of incubation.*

4b. (signal amplification) There are 3 kits we have used for tyramide-based signal amplification: (i) Dako TSA kit, (ii) Perkin-Elmer TSA kit, and (iii) Perkin-Elmer Cy3-TSA kit.

*We find that Dako has the greatest signal amplification capability. Perkin-Elmer works well for genes at mid-low levels of expression, but not as well as Dako for low levels of expression. Also, all kits are highly sensitive and should be shipped on ice and stored at 4C as extreme temperatures ruin the reagents (you may need to special request this when you order).*

1. Dako TSA kit
   1. Standard: add 2 drops of biotinylated tyramide amplification reagent (BTAR) to each slide, incubate ~15 min. at RT
   2. incubation time can be varied to get weaker or stronger signal
   3. kit also works well with a 1:10 (minimum) dilution of BTAR in TBS-T, 200 µL per slide incubated overnight at 4°C. This gives less background and works well with aged kits that give a weaker BTAR signal. For brand new kits can even try 1:20 dilution.
2. Perkin-Elmer TSA kit
   1. Standard: Dilute BTAR 1:50 with 1x dilution buffer. Add 150 µL per slide and incubate for ~7 min. at RT
   2. Vary the incubation time (from 3-15 min.) to vary the signal strength.
3. Perkin-Elmer Cy3-TSA kit
   1. Standard: Dilute 1:100 or 1:150 in amplification buffer and incubate at RT for ~10 min.
   2. Vary the dilution or incubation time to increase/decrease signal.
   3. This is a “one-step” amplification, as the TSA kit is already Cy3 conjugated (i.e. you do not need to use Cy3-SA in the following steps). As such, (i) and (ii) are more sensitive, but this works well to improve signal over no amplification at all.

5. Wash slides 4 times for 15 min. each in TBS-T at RT on a shaker.

6. Add secondary antibodies in TBS-T and incubate in the dark for 1-2 hours at RT.

*For (i) Dako TSA kit and (ii) Perkin-Elmer TSA kit from above, add Cy3 conjugated Strept-Avidin (Cy3-SA) at 1:500 in TBS-T, ~250 µL per slide. Incubate in the dark for 15-30 min. at RT. Biotin-SA interactions have a very strong affinity, so be careful with the incubation time. If you are using longer incubations for other secondaries, add the Cy3-SA ~20 min. before the end of those incubations. If you used additional primary antibodies, use the appropriate secondary antibody protocol here. For GFP, we use secondary Alexa 488 anti-rabbit or FITC anti-chicken. If secondary antibodies appear to be giving high levels of background, the slides can be re-blocked and secondaries can be diluted in 5% serum in TBS-T.*

7. Wash slides 4 times for 15 min. in TBS-T. We add DAPI on the second wash to stain all nuclei.

8. Rinse slides in water.

9. Mount using Biomedia Mounting Medium or a similar anti-fade mounting media.

***Biotin-labeled probe detection:***

1. Dilute streptavidin-HRP 1:100 in primary antibody diluent and add 150 µL per slide for 15 min. at RT

2. Wash in TBS-T 3 times for 10 min. each on shaker at RT

3. Signal amplification step. Same as #4b in the DIG detection protocol above.

4. Wash 3 times for 10 min. in TBS-T

5. For Dako TSA and Perkin-Elmer TSA, dilute Cy3-SA 1:500 in TBS-T and add 300 µL per slide. Incubate for 15-30 min. at RT.

6. Wash 3 times for 10 min. in TBS-T.

*Proceed directly to 11 if no additional antibodies will be used. RNA probe detection is now complete, so remaining steps are for detecting additional antigens using immuno-staining*.

7. Block slides at RT for 30-60 minutes in 10% serum in TBS-T

*see notes above under #1*

8. Add primary antibodies for other signal detection in 5% serum in TBS-T. i.e. for GFP as detailed in step #2 above.

9. Wash slides 4 times for 10 min. each in TBS-T at RT on a shaker.

10. Incubate with secondary antibody. i.e. FITC-anti-chicken or Alexa 488-anti-rabbit.

11. Wash slides 4 times for 15 min. in TBS-T. We add DAPI on the second wash to be able to additionally detect all nuclei.

12. Rinse slides in water.

13. Mount using Biomedia Mounting Medium.

**Recipes**

***4% Paraformaldehyde in 1x PBS (4% PFA) (200 mL)***

Heat 120 mL ddH_2_O to 65-70 C (1-2 min. in the microwave)

Add 8 g paraformaldehyde and mix in hood

Add 5 N NaOH until solution clears (~20 µL)

Add ddH_2_O to 150 mL

Add 50 mL of 4x PBS and mix well

Filter to remove particulates, cool to room temperature

pH to 7.0 if necessary and add ddH_2_O to 200 mL

Store at 4°C not more than 1 week

***10x Phosphate Buffered Saline (PBS) (1L)***

2.5g NaH_2_PO_4_-H_2_O

11g Na_2_HPO_4_-7H_2_O

90g NaCl

ddH_2_O to 1 L

DEPC treat and autoclave

We often use pre-made Sigma Phosphate Buffered Saline, pH 7.4 (1 foil pouch to 1L) (#P3813)

***PBS-triton***

.1% Triton X-100 in 1x PBS (1 mL of Triton per liter of 1x PBS)

10x PBS diluted in DEPC ddH_2_O

***0.1M TEA pH 8 (for acetylation) (1L)***

13.3 mL triethanolamine

983.7 mL DEPC dH_2_O

3 mL concentrated HCl (add to solution until pH is 8.0)

***Hybridization buffer (type I) (200 mL)***

100 mL formamide

50 mL 20x SSC

47 mL ddH_2_0

20 mg tRNA

20 µL Tween 20

DEPC ddH_2_0 to 200 mL

can be aliquoted and frozen at -20°C until use

***Hybridization buffer (type II)*** *[3]* ***(4 mL)***

2ml of 100% formamide

412 µL of H_2_O

480 µL of 5M NaCl

100 µL of 10% SDS

80 µL of yeast tRNA (10mg/ml)

80 µL of 50x Denhardt's solution

40 µL of 1M Tris.CL (pH 8.0)

800 µL of 50% Dextran Sulfate

8 µL of 0.5M EDTA

can be aliquoted and frozen at -20°C until use

***50x Denhardt’s solution***

5％ Ficoll Type 400

5％ Polyvinylpyrrolidone

5％ BSA

***Formamide wash buffer (500 mL)***

60 mL 5M NaCl (600 mM NaCl)

1 mL .5 M EDTA (1 mM EDTA)

5 mL 1M Tris pH 8 (10 mM Tris pH 8)

250 mL formamide (50% formamide) (add fresh just before use)

ddH_2_O to 500 mL

Buffer can be pre-made (ddH_2_O, NaCl, EDTA, Tris), aliquoted and frozen at -20°C. Pre-warm and add formamide prior to use.

***20X SSC (dilute to 2x and 0.2x before use) (1 L)***

175.3 g NaCl

88.2 g Na Citrate

Dissolve salts in 800 µL ddH_2_O

Adjust pH to 7.0 with 10 N NaOH

Add ddH2O to 1 L

DEPC treat and autoclave

We often use pre-made UltraPure 20x SSC buffer from Invitrogen (#15557-044)

***DEPC treatment***

Add 0.1% DEPC (diethyl-pyrocarbonate) in a hood (1 mL per L of solution)

Allow solution to sit at RT overnight and autoclave the following morning

DEPC reacts with amines so it cannot be directly added to Tris buffers. Make-up Tris with DEPC treated water and be very careful when pHing to avoid RNase contamination. Or use DNase/RNase free purchased water from Gibco (#4423).

***TBS-T***

0.1 M TRIS-HCl, pH7.5

0.15 M   NaCl

0.05% Tween- 20

***Blocking reagent in TNB buffer***

10 mL 1 M Tris pH 7.5 (.1M Tris pH 7.5)

3 mL 5M NaCl (.15M NaCl)

.5 g blocking reagent (.5% blocking reagent)

dH_2_O to 100 mL

Dissolve on hot plate with stirring (may take over an hour to dissolve). Aliquot and store at -20°C. Can be kept at 4°C for 24 hours.

***Additional reagents for making solutions***

ultra PURE water from Gibco #10977-015

ultraPURE 1M Tris-HCl pH 8.0 invitrogen #15568-025

ultraPure 10x TBE buffer invitrogen #15581-044

**References:**

1. Braissant O, Wahli W: **A simplified *in situ* hybridization protocol using non-radioactively labeled probes to detect abundant and rare mRNAs on tissue sections**. *Biochemica* 1998, **1**:10-16.

2. Kosman D, Mizutani CM, Lemons D, Cox WG, McGinnis W, Bier E: **Multiplex detection of RNA expression in Drosophila embryos**. *Science* 2004, **305**(5685):846.

3. Miyamichi K, Serizawa S, Kimura HM, Sakano H: **Continuous and overlapping expression domains of odorant receptor genes in the olfactory epithelium determine the dorsal/ventral positioning of glomeruli in the olfactory bulb**. *J Neurosci* 2005, **25**(14):3586-3592.

4. Zaidi AU, Enomoto H, Milbrandt J, Roth KA: **Dual fluorescent in situ hybridization and immunohistochemical detection with tyramide signal amplification**. *J Histochem Cytochem* 2000, **48**(10):1369-1375.
