# Supplementary material for: Lola regulates Drosophila olfactory projection neuron identity and targeting specificity
Source: Neural Dev. 2007 Jul 16;2:14. doi: 10.1186/1749-8104-2-14 (PMC1947980; doi:10.1186/1749-8104-2-14)
Supplement: Additional file 8 — Legends for supplemental Figures S1-S5. All supplemental figure titles and legends. [file 1749-8104-2-14-S8.doc]

Supplemental Figure Legends

Figure S1. Additional *lola* mutant allele MARCM analysis demonstrating dendritic targeting defects. **(a)** Representative images illustrating the dendrite phenotype of *lola^ore5D2^*^-/-^ (a strong hypomorphic allele caused by P-element insertion in the 5’ UTR), which is similar to that of the *lola*^-/-^ allele. adNB (a_1_), lNB (a_2_), vNB (a_3_), Mz19 adNB (a_4_), and Mz19 lNB (a_5_) clones are all affected. Single-cell DL1 clones are largely normal and target to DL1 (a_6_). **(b)** *lola^orc4-/-^* (*lola K^-/-^*) clones appear relatively normal and do not display the loss of targeting and ectopic targeting observed in clones of other mutant alleles. **(c)** *lola*^ore119-/-^  (*lola L^-/-^*) clones appear to have a subset of the null phenotypes. adNB (c_1_) and adSC (c_6_) clones seem unaffected by *lola L^-/-^*. lNB often show a loss of targeting to the VM1 and DA2 glomeruli, and a weak diffuseness of dendrites within the AL (c_2_). vNB clones often show a loss of DA1 targeting (c_3_). Mz19 adNB often show targeting of additional glomeruli (c_4_). Mz19 lNB show additional dendritic extensions to other regions of the AL (c_5_). Blue arrows or blue circles demark a loss of targeting to correct glomeruli. Yellow arrows or yellow circles denote ectopic targeting or dendritic extensions. Scale bar, 20 µM. anti-rat CD8::GFP in green, anti-mouse nc82 in magenta.

Figure S2. Additional *lola* isoform *in situ*s in the *Drosophila* brain. **(a)** Hypotheses prior to expression study. Different lineages of PNs may express different subsets of isoforms (I). Individual PNs may express different isoforms (II). No obvious isoform-specific expression in different PN classes (III). The goal of expression analysis was to distinguish between these possibilities. **(b)** Morphological labeling of typical pupal section including the salivary gland (SG), pupal case (PC), cuticle (Cu), eye disk (ED), wing disk (WD), optic lobe (OL), antenna lobe (AL), gut, brain, spiracle (Sp) and ventral nerve cord (VNC). **(c)** Morphological labeling of a 40x magnification of the 0h APF AL showing the location of the gut, neuropil (NP), antenna lobe dendrites (AL) and roughly outlined regions of the adPNs, lPNs and vPNs. **(d,e)** Verification of *in situ* protocols on pupal tissues. *drifter* (*dfr*) mRNA is expressed in lPNs but not adPNs (e), consistent with previous studies based on antibody staining. The sense control has little staining in the AL region, although non-specific staining is present in the neuropil (d). **(f-r)** *In situ* analyses of different *lola* isoforms. Sense probes show little specific signal (data not shown, see Figure 4a_2_-d_2_ for representative images). *In situ* analysis was performed for all *lola* isoforms as indicated, except J, M, O and R. All isoforms appear to have some expression in the region of the AL. However, punctate-staining and DAPI co-localization suggests that some isoform probes (i.e., see panels f, k, n, r) may be detecting pre-mRNA expression, or that some probes do not work as well as others. Some isoforms clearly have different domains of expression, for instance isoform N (p) has weak expression in the optic lobe (blue only region at the right of the image). White arrows point to GH146 positive PNs. White-dotted lines denote the rough boundary of the AL in these sections at 0h APF (d-r). DIG-labeled RNA probes in red, DAPI in blue, GFP in green.

Figure S3. Additional *lola* isoform RT-PCR analysis in the *Drosophila* brain. **(a)** A developmental timecourse of *lola* isoform expression in AL at 3^rd^ instar (red), 0h APF (black), 25h APF (green) and adult (cyan) stages suggests certain isoforms may be developmentally regulated in the AL. **(b)** A developmental timecourse of *lola* isoform expression in the optic lobe at 3^rd^ instar (cyan), 0h APF (black) and 25h APF (green) suggests *lola* isoforms are developmentally regulated in the optic lobe. **(c)** A comparison of unselected brain tissue (i.e. not labeled by Gal4-GH146), AL and optic lobe samples at 0h APF suggests that differences may exist in the expression of *lola* isoforms in different tissues. For all charts, individual *lola* isoforms are shown on the X-axis and relative abundance as compared to *actin42* is shown on the Y-axis. Error bars represent standard deviation from at least 2 independent samples, and samples were independently tested 5 times. Samples lacking error bars failed to produce product in 1 or more samples resulting in a single data point. Horizontal solid line represents a confidence limit of the average relative expression of samples near the detection limit based on CT values and reproducibility. Primer sets for isoforms C, E, F, G, H, I, J, M, Q, S and T span exons.

Figure S4. Effects of *UAS*-*lola* overexpression on adPN and lPN dendrites and axons. **(a)** Control adNB and lNB images. **(b-e)** Representative images of overexpression phenotypes for *UAS*-*lola A*, *UAS*-*lola L* and *UAS-lola T*. Transgenes and temperatures are as indicated. Expression of *UAS*-*lola L* at 25ºC (c) as well as *UAS-lola T* at 25ºC (d) or 29ºC (e) cause marked defects in adNB and lNB clones, mainly characterized by a large increase in dendrites in the AL that is difficult to quantify. Scale bar, 20 µM. anti-rat CD8::GFP in green, anti-mouse nc82 in magenta.

Figure S5. adNB and lNB phenotypes in *lola*^-/-^, *UAS*-*lola* MARCM clones. **(a)** Control images. **(b)** *lola*^-/-^ phenotype images. **(c)** Misexpression of *UAS-lola A* further disrupts adNB (c_1_) and lNB (c_2_) clones. Phenotypes observed in adPN and lPN neuroblast clones were highly variable. Expression of *UAS-lola A*, which had no phenotypes in a WT background, resulted in a marked reduction in dendritic branch extension and glomerular targeting as compared to *lola*^-/-^ clones. **(d)** *UAS*-*lola L* misexpression results in increased wandering dendrites in the AL and further disruption of normal glomerular PN targeting without affecting the number of labeled PNs. **(e)** Misexpression of *UAS-lola T* results in a dramatic reduction in the number of labeled cell bodies and a loss of dendritic mass and elaboration in the AL in all clone types. adPNs were hard to label, and clones often consisted of only 2-6 cells (instead of the normal 25-28). Dendritic elaboration was severely affected and it was difficult to discern if any glomeruli were actually innervated (Figure S4e_1_,e_2_). lPNs also had severe disruption of dendritic arborizations, and clone size was reduced to only 3-10 cells. Often cells within the clones appeared to be dying, perhaps explaining the reduction of cell numbers and the lack of observable dendrite and axonal extensions. Yellow arrows denote ectopic targeting. Blue circles denote a loss of normal glomerular targeting. Scale bar, 20 µM. anti-rat CD8::GFP in green, anti-mouse nc82 in magenta.
